# CONAN: A Tool to Decode Dynamical Information from Molecular Interaction Maps

**DOI:** 10.1016/j.bpj.2018.01.033

**Published:** 2018-03-27

**Authors:** Davide Mercadante, Frauke Gräter, Csaba Daday

**Affiliations:** 1 Interdisciplinary Center for Scientific Computing, Heidelberg University, Mathematikon, Heidelberg, Germany; 2 Molecular Biomechanics Group, Heidelberg Institute for Theoretical Studies, Heidelberg, Germany

## Abstract

The analysis of contacts is a powerful tool to understand biomolecular function in a series of contexts, from the investigation of dynamical behavior at equilibrium to the study of nonequilibrium dynamics in which the system moves between multiple states. We thus propose a tool called CONtact ANalysis (CONAN) that, from molecular dynamics (MD) trajectories, analyzes interresidue contacts, creates videos of time-resolved contact maps, and performs correlation, principal component, and cluster analysis, revealing how specific contacts relate to functionally relevant states sampled by MD. We present how CONAN can identify features describing the dynamics of ubiquitin both at equilibrium and during mechanical unfolding. Additionally, we show the analysis of MD trajectories of an *α*-synuclein mutant peptide that undergoes an *α*-*β* conformational transition that can be easily monitored using CONAN, which identifies the multiple states that the peptide explores along its conformational dynamics. The high versatility and ease of use of the software make CONAN a tool that can significantly facilitate the understanding of the complex dynamical behavior of proteins or other biomolecules. CONAN and its documentation are freely available for download on GitHub.

## Introduction

Protein dynamics critically determine biological function. Given how complex and nonisotropic proteins are, describing their dynamics is a challenging task. Contact maps represent a formidable instrument for depicting intramolecular or intermolecular interactions. By definition, they are two-dimensional (2D) matrices that are built employing a user-defined cutoff on the basis of a chosen interresidue distance criterion relevant to the investigated molecular behavior (1). They have been used to predict and describe the formation of protein complexes (2), to reconstruct the three-dimensional (3D) structure of proteins (3), in protein folding studies (4), or, more recently, as a base of Markov-state models for protein folding (5).

Contact maps encode all the structural information (secondary and tertiary structure) characterizing the investigated molecule. Helices can be identified by a thickening of the matrix diagonal, whereas parallel and antiparallel *β*-strands are represented as narrow contact stretches orthogonal or parallel to the diagonal of the matrix, respectively, and similar patterns can show parallel and antiparallel packing in general. Thus, contact maps encode structural information through an exhaustive 2D representation of distances.

However, molecular simulations additionally sample conformational properties of molecules through time. The incorporation of dynamical information in contact matrices is extremely valuable to describe structural changes observed along a trajectory. At equilibrium, the analysis of contacts along MD trajectories can be useful to understand the lifetime of contacts across the structure, the achievement of convergence, or new conformational states explored by a system (6, 7). Contact maps can also reveal information about the stability of intramolecular interactions during unfolding of the protein, as for example in force-probe molecular dynamics simulations (8). In these nonequilibrium conditions, the time-resolved analysis of contact maps can show the order in which contacts break along the pulling trajectory, which interactions are mostly responsible for withstanding the applied force, can identify unfolding intermediates, and whether there are multiple unfolding pathways.

Several tools have been created to visualize contact matrices and map the information they carry onto the 3D structure of molecules (3, 9, 10, 11, 12), but they mostly focus on encoding static (structural) information in them or require substantial input from the user. We here present a tool named CONtact ANalysis (CONAN) that, in addition to generating time-averaged quantities, also captures and encodes dynamical information from molecular dynamics (MD) trajectories into contact maps in a highly automated manner, requiring no scripting experience on the part of the user.

CONAN automatically generates publication-quality images and videos showing the evolution of contacts along MD trajectories and additionally provides a series of statistical analysis correlating the formation and rupture of contacts with time or with any observable of interest. It also implements contact map-based alternatives to commonly used techniques in computational biophysics, such as root-mean-square deviation (RMSD) and root-mean-square fluctuations (RMSFs), cluster analysis (in particular, hierarchical clustering (13)), and principal component analysis (PCA) (similar to (14)), thus offering the inherent advantage of avoiding the fitting procedure and/or the use of average coordinates required to perform these analyses based on atomic positions. CONAN also introduces an interresidue cross correlation based on the number of contacts rather than molecular fluctuations, again eschewing any interframe fitting.

We here report the analysis of test cases in which force-probe and equilibrium MD runs are performed on ubiquitin (15) and on a mutant peptide of *α*-synuclein (16), respectively. In addition, we illustrate how CONAN can also analyze protein-protein interactions by classifying villin headpiece homodimers (17) (Supporting Material). These test systems demonstrate the capabilities of CONAN in identifying conformational transitions. We show how the presented tool is helpful in the detailed description of an unfolding pathway of ubiquitin. The simulated *α*-synuclein fragment, on the other hand, undergoes an *α* to *β* transition that can be readily identified by CONAN. Contact maps colored by the chemical nature of the most long-lived interactions allow the straightforward identification of the chemical moieties playing a predominant role in the observed transition. Overall, the set of features comprised in CONAN make it a powerful tool for the analysis of MD simulations.

## Methods

We here restrict our discussion to the definition and choice of cutoffs. See Supporting Material and the online manual for a full explanation of possible outputs and methods.

### Definition of contacts

CONAN can be customized with up to three different cutoff distances:

$r_{\text{cut}}$ This is the main cutoff value. Any residue pair without any atoms within this cutoff is disregarded.
$r_{\text{inter}}$ This is the cutoff value under which interactions are formed.
$r_{\text{inter}}^{\text{high}}$ This is the cutoff value over which interactions are broken.

The main quantity CONAN uses is the interresidue distance defined as:

$$r_{ij}\left(t\right)=\{\begin{array}{cc} r_{ij}^{\text{min}}\left(t\right) & r_{ij}^{\text{min}}<\;r_{\text{cut}}\\ r_{\text{cut}} & r_{ij}^{\text{min}}\geq r_{\text{cut}}, \end{array}$$

where $r_{ij}^{\text{min}}\left(t\right)$ is the minimum distance between atoms from residues *i* and *j*. The user can choose a given class of atoms of interest (C_*α*_, backbone atoms, side chains, any heavy atoms, any atom, etc.). In the analysis presented below, we always consider only heavy atom distances, as it reduces the search space by a factor of ∼4 without losing significant information, a main cutoff $r_{\text{cut}}=1$ nm, and interactions defined using the same cutoff, $r_{\text{inter}}=r_{\text{inter}}^{\text{high}}=0.5$ nm. The approach of using two different cutoffs for breaking and forming interactions, proposed before by Best et al. (18), avoids the underestimation of the stability of interactions due to a few outlier frames. However, in this case, we are using only one value for simplicity.

### Functionality of CONAN

**Figure 1:**
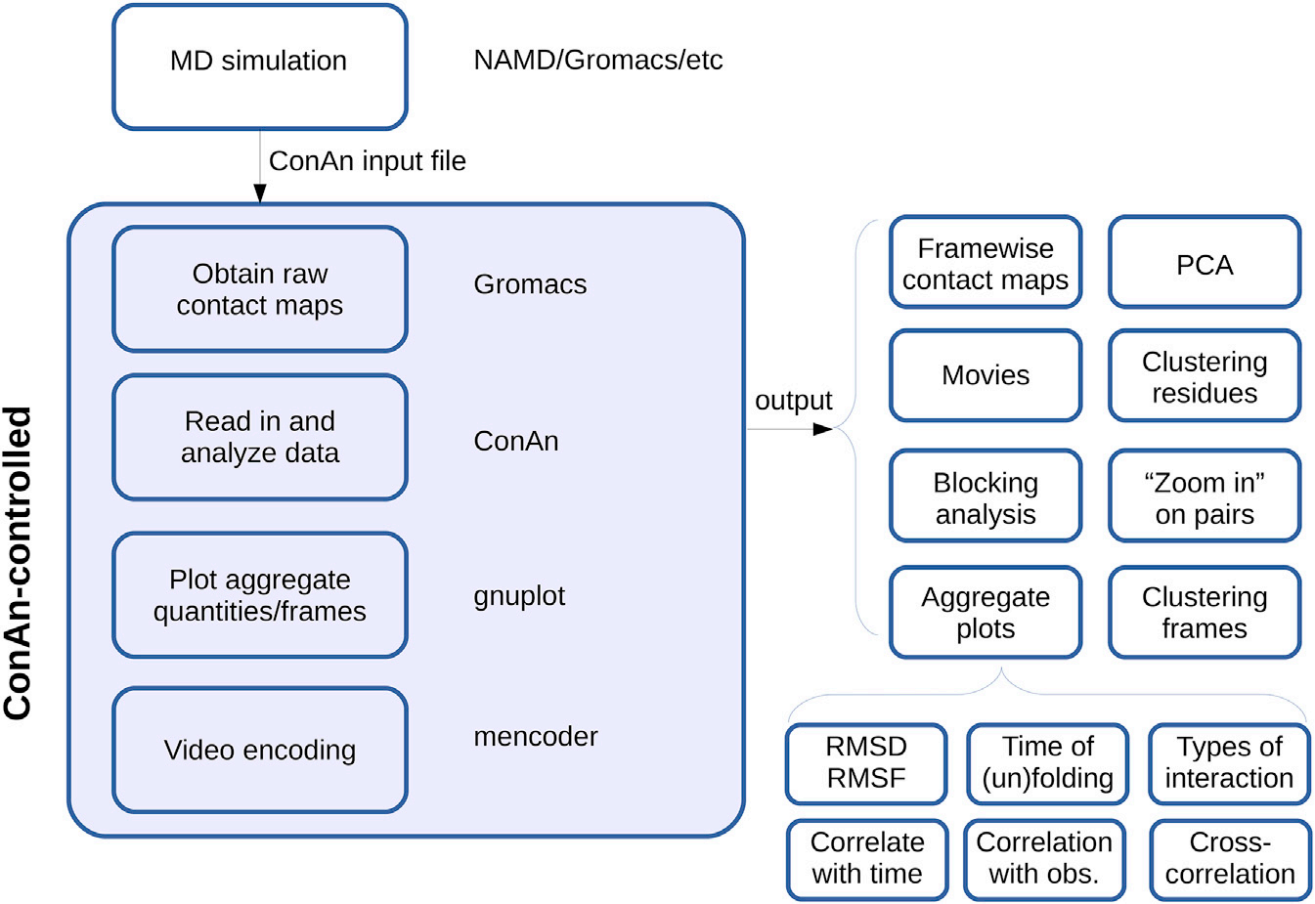
Flowchart illustrating CONAN’s main workflow. The flowchart shows the different tools (left-hand side of the figure) used by CONAN to output a series of analysis required by the user (right*-*hand side of the figure). The kinds of analysis to perform, as well as the corresponding parameters, are defined in the CONAN input file. All of the plots except this figure and Fig. 4*A* are based on standard outputs from CONAN, augmented mainly only by 3D structures and highlights. obs., observation. To see this figure in color, go online.

CONAN has been designed with the purpose of being both user-friendly and versatile and requires few extra packages installed. To run the contact-based analysis of the performed MD simulations, the user provides a single input file with a set of keywords that the software interprets to perform the desired analyses. The input files used in the example cases used here are in the Supporting Material. To build contact maps, CONAN utilizes the mdmat utility provided in the GROMACS MD simulation engine (19) and interprets the output to retrieve the required statistics (Fig. 1). Using gmx mdmat has the inherent advantage of being compatible with other GROMACS tools (for example, using the custom index files). We stress, however, that a series of tools are available to convert trajectories to GROMACS format, and therefore CONAN is also compatible with results obtained from other packages. In the Supporting Material, we illustrate this by analyzing a trajectory obtained from CHARMM (Accelrys, San Diego, CA).

CONAN is programmed in Python 3 and is therefore easily available as a package as well. Given the possibly costly memory requirements of $\propto N_{\text{res}}^{2}\times N_{\text{frames}}$ (number of residues squared times number of frames), we took particular care of economical data storage. All of the memory-sensitive quantities are stored in a sparse way and using half-precision (numpy’s float16 structure) for efficient memory use. Furthermore, CONAN can be run in an “economy mode” in which only one frame and only aggregate quantities are stored, thereby reducing the memory usage to a minimum. Analogously, since the plain-text matrices can occupy significant disk space, the user also has an option to only store aggregate ones and/or only the .png files. Further information on requirements can be found in the manual. The current main bottleneck is generating the framewise .png files in gnuplot, and the CONAN-specific commands are executed very efficiently through vectorized instructions implemented in numpy and scipy. For example, turning off .png generation, the given example of ubiquitin unfolding, involving 1976 pairs and 2357 frames, requires ∼33 s on a single core of an Intel i7-4770 (Intel, Santa Clara, CA) central processing unit for a standard analysis, and 84 s for a full analysis (correlations, both types of cluster analysis, and PCA); in fact, even these timings are mostly input/output-limited.

CONAN uses gnuplot (20) to plot at each time step the interaction maps calculated from the contact matrix and collates them using video encoding (×264 in this case) for the creation of videos that show time-resolved evolution of the contacts. As color schemes for the creation of contact maps, we use cubehelix (21) for distances and times (sequential data) and an 11-class version of Cynthia Brewer’s PuOr (purple-orange) scheme (22) for divergent data (changes or correlation coefficients), in which we set the central point to be exactly white (#FFFFFF in hexadecimal). CONAN, its documentation, and example use cases are freely available for download at https://github.com/HITS-MBM/conan under GPL 3.0. Further examples and explanations can be found on http://contactmaps.blogspot.com.

## Results and Discussion

### Ubiquitin equilibrium dynamics analyzed with CONAN

Ubiquitin is a small molecule structurally characterized by different secondary structure elements (one *α*-helix and a four strands *β*-sheet), and a useful model system for computational and experimental studies investigating protein structural and dynamical behavior: from the investigation of protein folding (23) to the testing of protein force fields used in MD simulations (24). We have herein chosen this molecule to provide a test case representing the capabilities of CONAN and therefore have performed equilibrium and force-probe MD simulations to illustrate how contact map analysis, as provided by CONAN, can lead to a deep understanding of protein functional dynamics.

**Figure 2:**
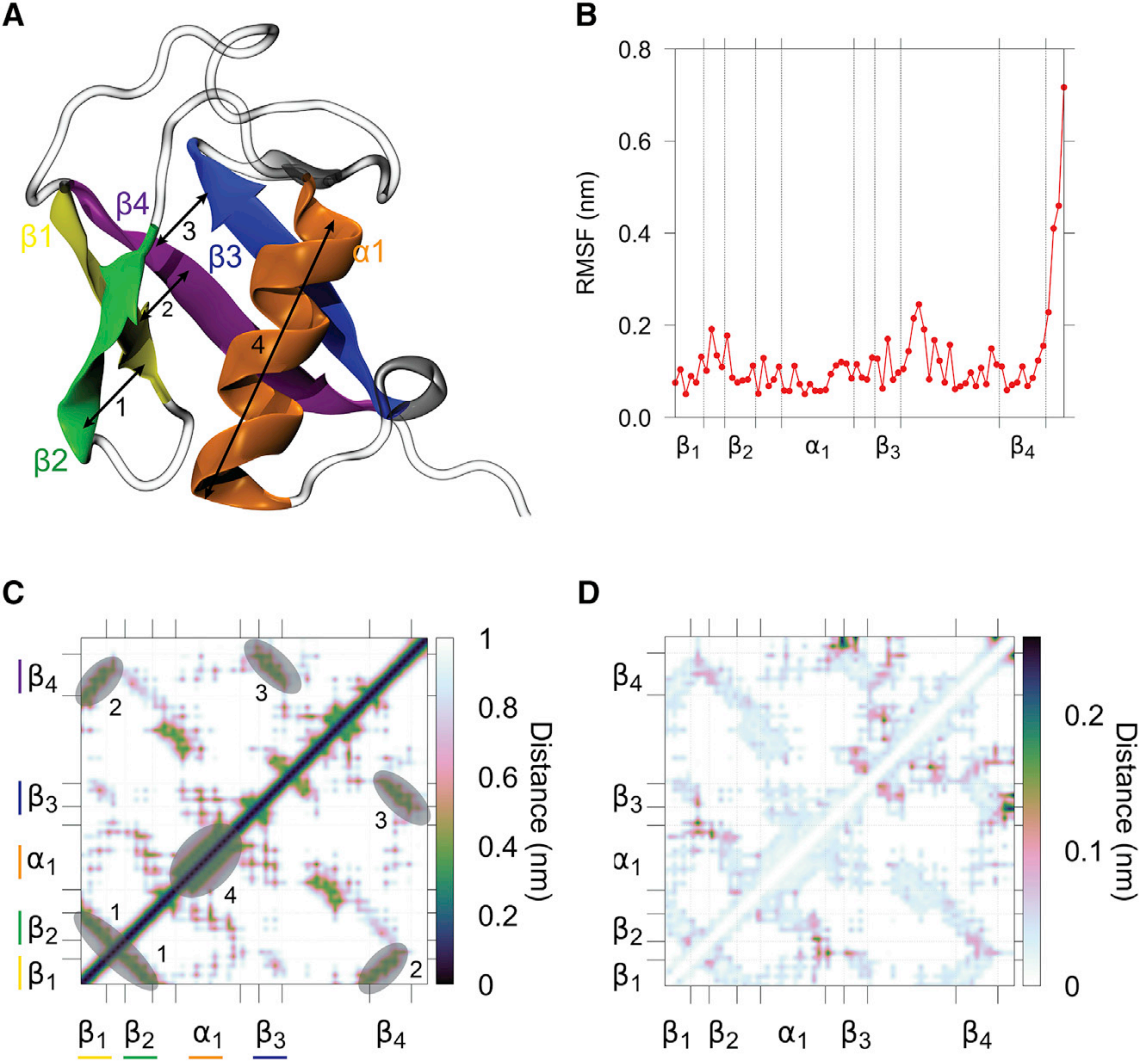
Dynamics of ubiquitin at equilibrium investigated by contact maps. (*A*) The structure of a ubiquitin molecule in a cartoon representation and colored according to its secondary structure elements and numbers denoting the most important groups of contacts are shown. (*B*) 3D coordinates-based root mean-square fluctuations (RMSFs) of ubiquitin are shown. (*C*) The average contact map is shown, with circles highlighting the most important contacts. (*D*) A symmetric contact map showing the standard deviation of contact distances along the trajectory. To see this figure in color, go online.

At equilibrium, we computed the average contact map of the protein (Fig. 2 *C*), which clearly shows that the protein’s secondary structure is stable, with the $\alpha_{1}$ helix, the antiparallel packing between strands $\beta_{1}$-$\beta_{2}$, $\beta_{3}$-$\beta_{4}$, and the parallel packing between strands $\beta_{2}$-$\beta_{3}$ and between $\beta_{1}$-$\beta_{4}$. Furthermore, we can also observe fluctuations between interresidue distances (Fig. 2 *D*), which are dominated by the motion of the C-terminal linker with respect to the $\beta_{3}$ strand and its immediate vicinity. RMSFs (Fig. 2 *B*) are a widely used observable to monitor, on average, the dynamics of residues within a structure. CONAN’s interresidue distance fluctuations can be considered as more reliable than RMSF values, especially when large fluctuations occur, as average positions can be physically meaningless. Furthermore, the output contains the most relevant interactions (changing only within values lower than the main cutoff) rather than identifying single residues.

**Figure 3:**
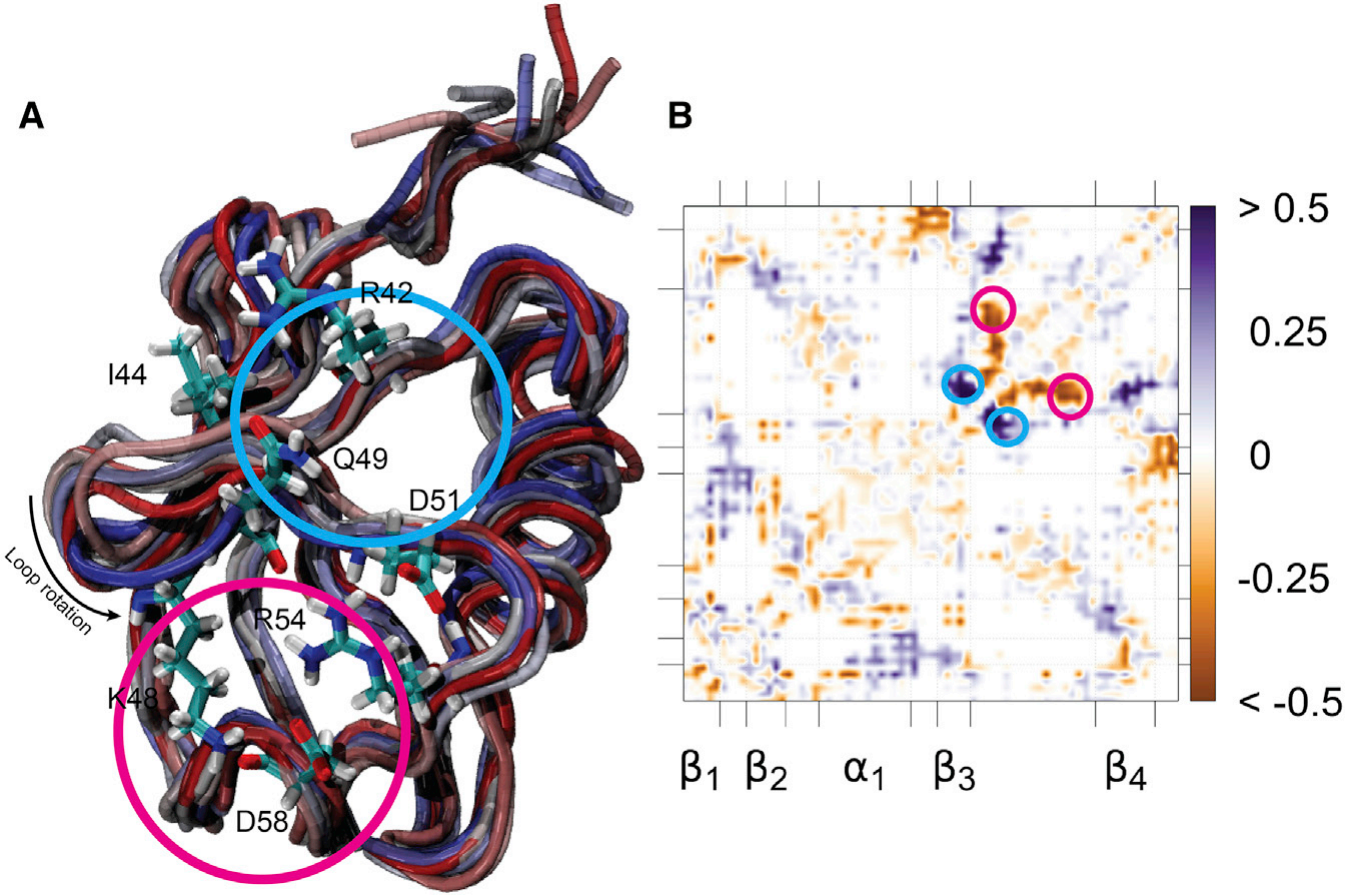
Correlation of interresidue distance with time. (*A*) An ensemble of ubiquitin at the equilibrium is shown. The conformers shown have been collected at every 5 ns and are colored as a function of time from red (start of the simulation) to blue (end of the simulation). The residues involved in pairs showing the highest correlations with time in map (*B*) are shown in licorice. (*B*) Correlation of interresidue distances with time are shown. Negative Pearson coefficients (*orange*) correspond to contact formation and positive ones (*purple*) correspond to contact ruptures. We highlight the largest changes in blue (ruptures) and pink (formations) in both plots.

The correlation of interresidue contacts with time shows a significant conformational rearrangement of the protein (Fig. 3 *A*). In particular, residue K48 drifts and increases its distance from residues I42 and R44 and approaches residue D58 with which it establishes a stable salt bridge. The salt bridge network newly formed between D51, R54, D58, and K48 is responsible for this stable conformation observed at the end of the simulated trajectory and representing a stable conformational state of the protein (Fig. 3). The correlation map clearly pinpoints the relevant regions in the 3D overlap of structures (Fig. 3 *A*). The conformational rearrangement is also captured in the interresidue cross correlation, which identifies a competitive behavior between R42 and D58 (see Fig. S1 and further explanation there).

This kind of analysis can be useful to diagnose drifts from the starting structure (for example, a crystal structure or a homology model), which might also reflect a lack of convergence in the simulation: in the (usually not achievable) limit of full exploration of the phase space, none of the interresidue or interdomain distances would show a significant correlation with time. In this case, a relatively short simulation time (50 ns) has been chosen, as can be seen by the significant time correlations identified. CONAN interestingly resolves a “local drift,” with some regions equilibrated and others still changing conformation; this would be quite difficult to define in terms of 3D coordinates.

As mentioned in the Methods, CONAN can also identify the sequence determinants of macroscopic quantities; we show how certain loop rearrangements explain changes in radius of gyration $R_{g}$ in Fig. S2.

### Unfolding trajectory of ubiquitin

Beyond studying the behavior of molecules at equilibrium, nonequilibrium trajectories can also be analyzed by looking at the formation or rupture of contacts. In this case, we show how the unfolding of ubiquitin, triggered by the application of an external force, can be mapped onto the protein’s structure through the use of CONAN.

**Figure 4:**
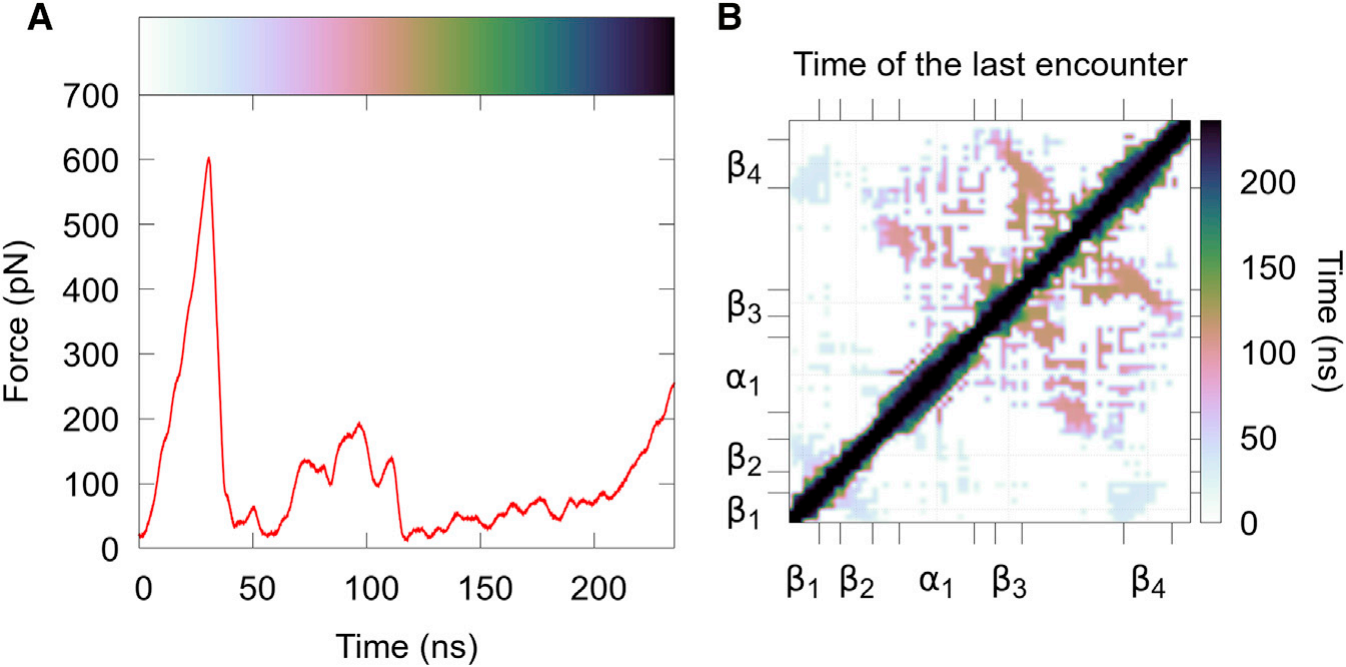
Force-driven unfolding of ubiquitin. (*A*) A force profile as a function of time is shown. (*B*) A time-encoded contact map showing the times of contact breaking is shown. The color code of subplot (*B*) is repeated in (*A*) for ease of comparison. To see this figure in color, go online.

Fig. 4 *B* reports the last encounter of residue pairs (i.e., unfolding times) and shows how these events are clearly clustered around two moments. First, around 40 ns (*faint gray*), $\beta_{1}$ loses contact with $\beta_{4}$ and $\beta_{2}$. Then, around 100 ns (*pink-orange*), $\alpha_{1}$ and $\beta_{3}-\beta_{4}$ lose contact with each other. Both of these events coincide with a drop in force (at ∼40 and ∼100 ns), reflecting barrier crossing events (Fig. 4 *A*). Finally, at various points in time after ∼150 ns (*green or darker*), $\alpha_{1}$ and various small helical regions lose contact as the peptide becomes straighter. This last part of the unfolding happens without any resistance; therefore, the events happen without any cooperativity. The above-described pathway is in close agreement with previous MD studies of ubiquitin under force (25). Fig. S4 contains the differential contact maps of the four stages. Finally, the different domains of the ubiquitin unfolding can also be detected by residue clustering, clearly distinguishing the two *β*-strands from the other residues (see Fig. S5).

In short, Fig. 4 *B* summarizes an entire trajectory in a simple, intuitive 2D image. This object can further be used to cluster different independent unfolding trajectories either visually or through rigorous methods (see Supporting Material for an example of this).

### *α*-Helix to *β*-strand transition of a mutant peptide from *α*-synuclein revealed by contact analysis

*α*-Synuclein is a well-studied disordered protein associated with the occurrence of Parkinson’s disease (26, 27, 28). As in the case of other neurodegenerative disorders mediated by peptides, the molecular basis of the disease is related to a profound structural transition from an *α*-helix to a *β*-sheet, which is an aggregation-prone state (29, 30). The mutation of glutamate to lysine at position 46 along the sequence (E46K) accelerates cytotoxicity of *α*-synuclein inside cells (31, 32, 33, 34). We have chosen this system to show that CONAN can readily identify the pathological conformational transition from *α*-helix to *β*-strand. The simulations, performed in replicates of 1 *μ*s, repeatedly show an abrupt conformational change from a starting *α*-helical conformation into a random coil and finally into a *β*-hairpin.

**Figure 5:**
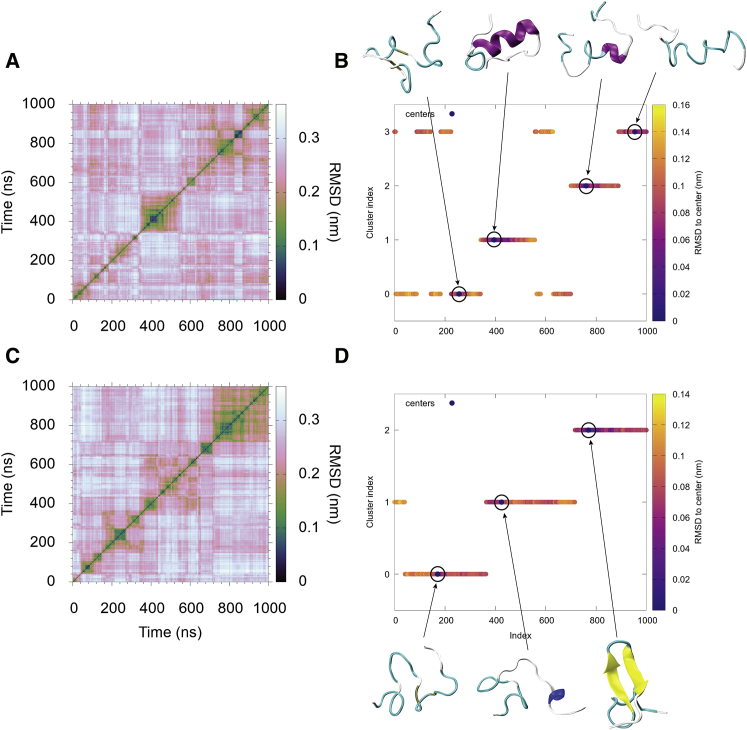
Contact-based cluster analysis of the wild-type and mutated (E46K) form of *α*-synuclein investigated using CONAN. (*A*) and (*C*) show the inter-frame RMSD of the trajectories (see Methods for details) describing the conformational dynamics of the simulated peptides. (*B*) and (*D*) show the clustering obtained for the simulated ensemble. The graphs describe the time evolution of clusters with points colored according to the distance of each conformation from the cluster medoid (*circled*). The 3D structures for each medoid color-coded by secondary structure are shown on the right-hand side of the figure. To see this figure in color, go online.

The cluster analysis on the trajectories of the simulated *α*-synuclein fragments reveals different behaviors for the wild-type and the E46K mutant. In the case of the wild-type, the fragment oscillates between three states defined by clusters 0, 1, and 3, which are mostly *α*-helical, while cluster 2 is scarcely populated and features a shorter helix and a more molten-globule configuration. The E46K mutant, on the other hand, shows a time-resolved evolution of the states leading to the formation of a *β*-hairpin, which is characteristic of intermediates assembling in fibrillary supramolecular structures (Fig. 5). We pinpoint the specific interactions responsible for these differences using CONAN (Figs. S6 and S7). The dendrograms of the wild-type and the mutant fragment can be found in Figs. S8 and S9.

## Conclusions

Biological function is ultimately linked to molecular structure and its motions. The analysis of molecular contacts is exceptionally useful to map particular dynamic events that characterize the physiological or pathological behavior of macromolecules onto a molecular structure. Although bearing a plentiful amount of information and being extensively used to visualize static information (crystal structures or averages over the simulated time), contact maps have not been widely employed to visualize or interpret dynamical information. Here, we developed a tool that constructs time-resolved contact maps and also performs statistical, principal component, or cluster analyses to extract evidence of how specific contacts contribute to the overall observed dynamics of a macromolecule. CONAN is a tool developed with the purpose to combine the analysis of contacts to a series of analytic frameworks and to provide a highly automated instrument for a varied analysis of contacts with respect to the conformational ensembles explored by MD simulations. With the use of a single input file, CONAN computes contact maps and provides a range of publication-grade figures and videos that are automatically generated and show how specific contacts are responsible for an investigated behavior.

Interestingly, the analysis of unfolding trajectories by means of monitoring formation and rupture of contacts can not only reveal contact rupture but also the formation of new and variably transient contacts. Such analyses can be applied to understand folding of molecules in which exhaustive sampling is provided and can, for instance, reveal the formation of local element structures along a folding pathway (24, 25) to reveal cooperative effects in protein-coupled binding and folding processes (35) or critically functional allosteric and catalytic events along protein structures (36). We believe that the highly automated output creation and the simplicity of the user interface is key to a widespread use of the contact-based analyses presented, which can enhance the understanding of molecular systems far beyond the test cases presented here.

## Author Contributions

D.M. and C.D. performed the simulations and data analysis. All three authors planned and wrote the manuscript. C.D. conceived of and wrote the software.

## Supporting Material

Document S1. Supporting Materials and Methods and Figs. S1–S17

Movie S1. Ubiquitin Unfolding: Framewise Contact Maps

Movie S2. Ubiquitin Unfolding: Framewise Differential Contact Maps with Regard to Initial Frame

Movie S3. Ubiquitin Unfolding: Framewise Differential Contact Maps with Regard to Previous Frame

Movie S4. *α*-Synuclein Fragment: Wild-Type

Movie S5. *α*-Synuclein Fragment: E46K Mutant

Document S2. Article plus Supporting Material
