# Supplementary material for: CONAN: A Tool to Decode Dynamical Information from Molecular Interaction Maps
Source: Biophys J. 2018 Mar 27;114(6):1267–73. doi: 10.1016/j.bpj.2018.01.033 (PMC5883949; doi:10.1016/j.bpj.2018.01.033)
Supplement: Document S1. Supporting Materials and Methods and Figs. S1–S17 [file mmc1.pdf]

Biophysical Journal, Volume *114*

Supplemental Information

CONAN: A Tool to Decode Dynamical Information from Molecular Interaction Maps

Davide Mercadante, Frauke Gräter, and Csaba Daday

Supporting Information for:
CONAN: a tool to decode dynamical information from molecular interaction maps

D. Mercadante and F. Gräter and C. Daday

January 19, 2018

Abstract

1 Methods

1.1 Molecular dynamics simulations

A molecule of ubiquitin [1] and a fragment of α-synuclein extracted from a complex with an engineered protein [2] (PDB codes: 1UBI and 4BXL respectively) were placed in the center of dodecahedron boxes having volumes so that the shortest distance between periodic images was at least 3 nm. Hydrogen atoms were added and a topology was created by assigning to each particle parameters from the AMBER03 force field [3]. After topology creation, the proteins were energy minimized for 20000 steps using a steepest descent algorithm with a force tolerance of 20 kJ $mol^{-1}nm^{-1}$ and a step size of 0.001. The system was then solvated with TIP3P water molecules [4]. Sodium (Na^+) and chloride (Cl^-) ions were added in order to reach an ionic strength of 0.15 M. After solvation and insertion of ions, the systems were firstly equilibrated in the NVT ensemble in which particles' velocities were generated according to a Boltzmann distribution obtained at 300 K. In the NVT step, which was carried out for 500 ps, the temperature was kept constant at 300 K using a V-rescale thermostat [5]. Subsequently to the NVT step, equilibration was continued in the NpT ensemble for additional 500 ps, through which temperature and pressure were kept constant at the values of 300 K and 1 bar respectively. As for the equilibration in NVT ensemble, temperature was kept constant by using a V- rescale thermostat with a time constant of 0.5 ps, whereas pressure was coupled through a Parrinello-Rahman barostat [6] in all dimensions (isotropic pressure coupling) with a time constant of 2.0 ps and a compressibility of $4.5 \cdot 10^{-5}$ bar^{-1}. During both NVT and NpT steps, a restraint on the positions of all the atoms of the proteins was applied using an external harmonic potential of 1000 kJ $mol^{-1}nm^{-1}$.

Production runs were then carried out in the NpT ensemble using the previously defined parameters for pressure and temperature for 50 and 1000 ns for ubiquitin and the α-synuclein fragment respectively. The GROMACS 5.1.2 [7] package was used to perform the MD simulations, whereas VMD 1.9.1 and Tachyon [8, 9] and UCSF Chimera packages were used to visually analyze the trajectories and render images respectively. Chimera is developed by the Resource for Biocomputing, Visualization, and Informatics at the University of California, San Francisco (supported by NIGMS P41-GM103311).

We received a trajectory of dense solution of 8 villin headpiece chains from Michael Feig et al. [10] for analysis with CONAN. It was obtained with CHARMM [11] through OpenMM. The solution has an effective density of 32 mM, the villins were neutralized with Cl^- ions. The CHARMM 36 force field was used for it with TIP3P water and an increased water-protein interaction by a factor of 1.09 [12]. The simulation was performed in the NVT ensemble with a cubic box with a side of about 7.4 nm. The trajectory has a length of 2μs using a 2 fs time step and a Langevin thermostat with a friction coefficient of 0.01 ps^{-1}. The particle-mesh Ewald method was used for Coulomb interactions.

1.2 Force-probe molecular dynamics simulations of ubiquitin

The molecule of ubiquitin in the conformation extracted at the end of the equilibrium simulation, was placed in a rectangular box of dimensions $28 \times 7 \times 7$ nm for force-probe MD simulations, where the centers of mass of the N- and C-terminal residues were aligned with the longest semi-axis. The protein was placed at the center of the box and after the NVT and NpT equilibration steps mentioned above, it was pulled at its N- and C-termini with the first and the last residues being considered as pulling groups. Center of mass pulling was performed on these groups by applying a harmonic potential to achieve constant velocity pulling at the speed of 0.1 ms^{-1} and a force

constant of 500 kJ $mol^{-1}nm^{-2}$. The pulling was directionally applied along the longest semi-axis of the box and in opposite directions for the two pulling groups. The simulations were stopped after 235.6 ns, when secondary structure elements of ubiquitin were fully lost.

1.3 CONAN output and specific methods

1.3.1 Basic output

The output of CONAN is a set of plain-text files and images. The advantage of plain-text over more compact forms is the ease of a quick comparison and filtering right in the terminal (through tools like awk, sort, diff, grep).

1.3.2 Further classes of output

A number of advanced features are available in CONAN: principal component analysis [13], hierarchical clustering of contact maps (classifying frames from a trajectory), hierarchical clustering of residues (classifying residues and detecting domains), and blocking analysis [14] (detecting the correlation time of contact maps). The same classes of analysis are also available for the analysis of asymmetric data, i.e., contacts formed between chains or domains. In Section 6, we show an analysis of protein assembly as an example.

1.3.3 Correlation contact maps

CONAN can compute four kinds of correlations to extract dynamical information (PCC(A(t), B(t)) is the Pearson correlation coefficient between vectors A and B, commonly known as the "r-value"):

PCC($r_{ij}(t)$, t) : time-correlation of inter-residue distance, identifying drifts.

PCC($r_{ij}(t)$, A(t)) : correlation of inter-residue distance and observable A, identifying sequence determinants.

PCC($r_{ij}(t)$, $r_{ab}(t)$) : correlation of inter-residue distance and a specific distance between a pre-defined pair of residues a and b. This is in the "zoom" module. Any number of such pairs ab can be specified.

PCC($N_i(t)$, $N_j(t)$) : inter-residue cross-correlation of the weighted number of contacts $N(t)$.

The "weighted number of contacts" is defined as:

$$N_i(t) = \sum_j (1 - \frac{r_{ij}(t)}{r_{\text{cut}}}).$$

This cross-correlation is a fitting-free alternative to the commonly used "dynamic cross correlation" of deviations from average positions. Note that all of the vectors have the same length, which is the number of frames in the simulation: "missing values", i.e., the distance between two residues that move outside the main cutoff radius, are replaced by the main cutoff radius.

1.4 Hierarchical clustering

CONAN uses Ward's agglomerative hierarcical clustering algorithm [15] to divide residues of a protein or frames of a trajectory into clusters, each with a representative data point (residue or frame) called medoid. Users can opt to interactively choose the desired number(s) of clusters, or to pre-define this number. The dendrogram, for ease of visibility, only displays the top 100 nodes if there are more than 100 data points. The medoid is defined after the clustering is complete by finding the data point with the lowest average distance to data points in its own cluster.

1.4.1 Frames

For the clustering of frames (conformations) composing a trajectory, we use the inter-frame RMSD as the distance metric. Between a conformation at t_1 and t_2, the distance will be given by:

$$\text{RMSD}(t_1, t_2) = \frac{1}{N_{\text{res}}} \sum_{i,j} \Delta r_{ij}(t_1, t_2)^2.$$

1.4.2 Residues

For the clustering of residues, we use the following three metrics between two residues i and j:

1. Average distance in real space:

$$d_{ij} = \bar{r}_{ij}$$

This is a straightforward criterion that is however still a better choice than the distance in the average structure since it does not involve a fitting procedure.

2. Total interaction time:

$$d_{ij} = 1 - \frac{t_{ij}^{\text{inter}}}{t^{\text{total}}}$$

where we use the interaction time between i and j and subtract this from 1 to have high numbers corresponding to low distances. This can be similar to the quantity calculated in Eq. 1, but it can be more representative of actual physical interactions.

3. Inter-residue cross-correlation:

$$d_{ij} = 1 - \text{PCC}(N_i(t), N_j(t))^2,$$

where $\text{PCC}(N_i(t), N_j(t))$ has been described before (Section 1.3.3). This correlation is squared to ameliorate the effect of spurious correlations.

1.5 Principal component analysis

CONAN can be used to do principal component analysis (PCA) on inter-residue distances. Therefore, it uses internal rather than Cartesian coordinates to perform a principal component analysis. Interestingly, the use of internal versus Cartesian coordinates has been recently investigated showing that even though Cartesian coordinates may capture overall motions of a protein, internal coordinates more accurately catch the underlying free-energy landscape of protein dynamics [13].

PCA is implemented across all the residue pairs that ever come closer than the main cutoff. Supposing that there are M such residue pairs, where $M \leq (N_{\text{res}}(N_{\text{res}} - 1)/2)$ if there are N_{res} residue pairs, CONAN computes and diagonalizes the $M \times M$ covariance matrix and prints out a user-defined number of eigenvectors and projections in order of eigenvalues. The time evolution of a residue pair distance will then be given by:

$$r_{ij} = \bar{r}_{ij} + \sum_{u}^{M} c_u^{ij} x_u(t) \approx \bar{r}_{ij} + \sum_{u}^{M_{\text{cut}}} c_u^{ij} x_u(t),$$

where the last approximation shows the often-used truncation of only the first few (in this example, M_{cut}) projections, c_u^{ij} denote the coefficients of principal component u on residue pair ij, and $x_u(t)$ is the time evolution of the projection onto principal component u.

1.6 Asymmetric contact maps

CONAN can do the above analyses on asymmetric cases, i.e., monitoring contacts between one range of residues and another one. This can be a basis for analyzing an interaction between domains or between protein-protein chains.

We will analyze villin-villin interchain docking in Section 6. In the special case of dimeric docking, such as this one, CONAN also takes into account the fact that the order of chains is arbitrary. It sorts dimers by preferring poses in which the N-terminus of the first chain is closer to the C-terminus of the second chain. It does this by comparing the "weighted number of contacts" in both the upper and lower triangles of the inter-chain contact map, and transposing the contact map if the lower triangle is found to have shorter distances than the upper one.

2 Equilibrium simulations: ubiquitin

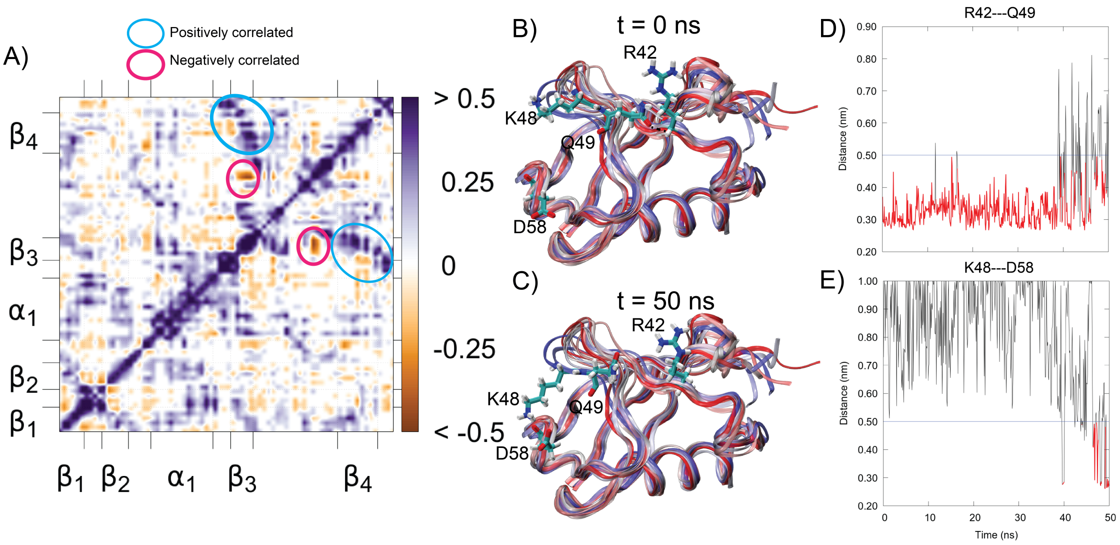

Figure S1: Inter-residue cross-correlation reveals competitive interactions. (A) Inter-residue cross-correlation between the weighted number of contacts of residues in ubiquitin. Particularly low and high values are highlighted in pink (negative cross-correlation, i.e., competitivity) and blue (positive cross-correlation, i.e., cooperativity). The pink circle highlights a competitivity between R42-D58, despite the fact that these residues are well outside each other's radii of interactions. This can be explained by the breaking of a contact between R42-Q49 (shown at t=0 ns in (B) and the time evolution is shown in D) and the forming of a salt bridge between K48-D58 (shown at t=50 ns in C and the time-evolution shown in E).

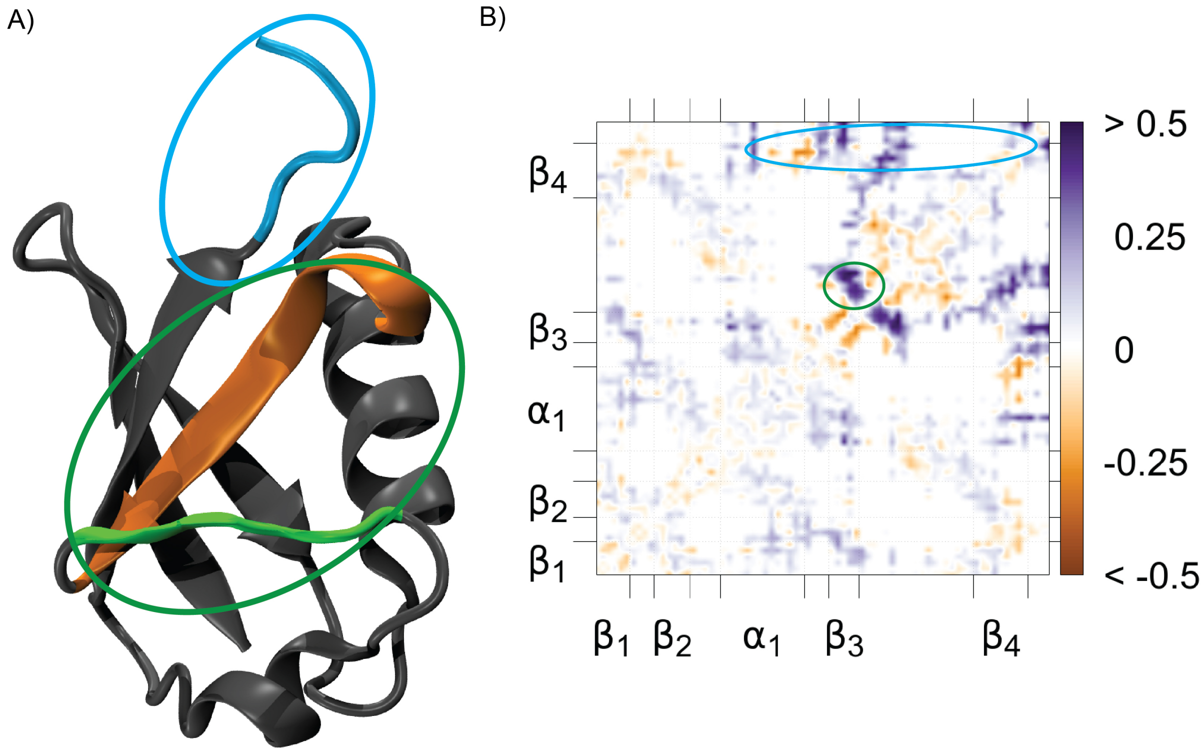

Figure S2: Pearson's correlation of the contact distances with radius of gyration sampled for ubiquitin. (A) Structure of ubiquitin highlighting the regions that show the highest positive correlation between inter-residue distances and the radius of gyration sampled during the simulations. (B) The symmetric correlation map shows positive and negative correlation (Pearson's correlation coefficient) in purple and orange respectively. there are two major regions of contacts that explain increases in the radius of gyration. Firstly, whenever the C-terminal flexible linker loses contacts with the rest of the protein, this increases the radius of gyration, and secondly, the distances between residues in the flexible linker and strand β_3 also positively correlate with R_g. In both cases, the correlation involves the distancing of a flexible linker from the rest of the protein structure, i.e., the average distances from the center of mass of the entire structure will tend to increase, thereby increasing the radius of gyration.

3 Unfolding simulations: ubiquitin

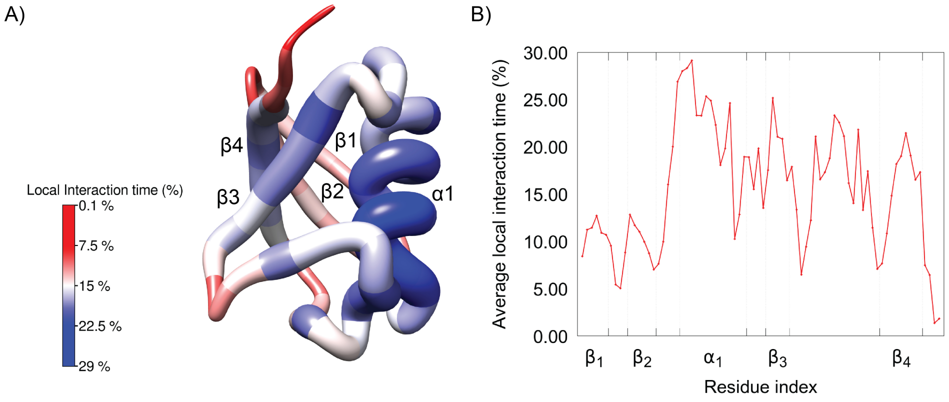

Figure S3: Local interaction time calculated for the unfolding trajectory of ubiquitin. (A) Structure of ubiquitin showing, qualitatively, the local interaction lifetime measured in the unfolding pathway that is quantitatively shown in (B) as a function of the protein's sequence. The structure has been colored according to the "local interaction time". The radius of the ribbons is proportional to this observable, quantitatively reported in the color palette on the left hand side of the figure. Regions in red correspond to parts of the protein that have the longest-lived interactions, i.e., unfold last.

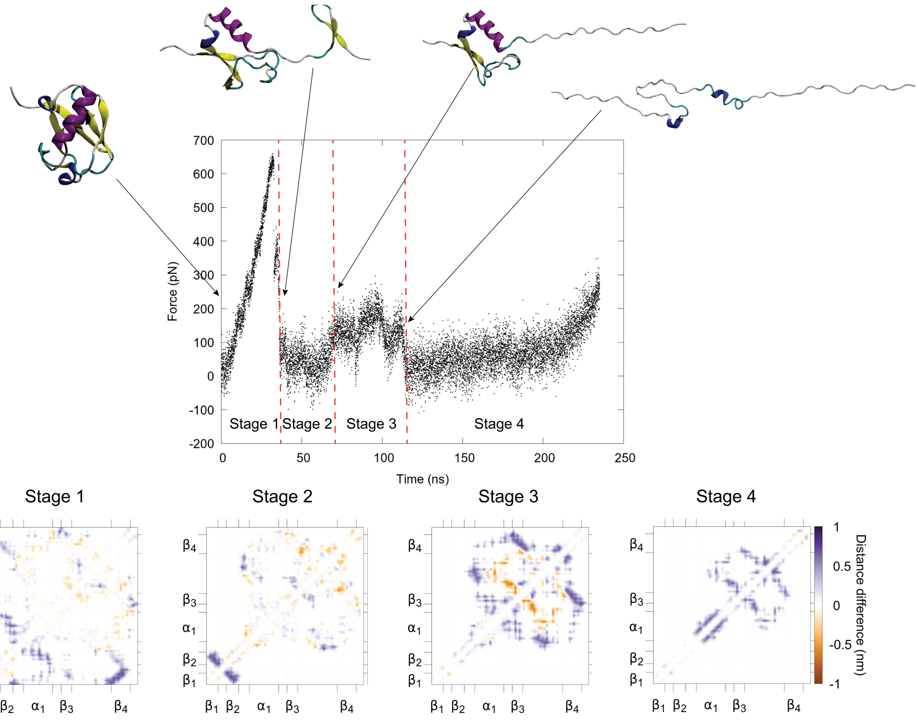

Figure S4: Force-driven unfolding of ubiquitin analyzed through differential contact maps. The force profile as a function of time of ubiquitin is shown in the top panel and divided into four distinct stages (Stages 1 to 4) following clear trends in the evolution of the force withstood by the molecule. For each stage, representative conformations are shown using a cartoon representation and colored by secondary structure. α-helices are colored purple, 3-10 helices blue, β-strands yellow, β-turns cyan, and loops white. In the lower panel, differential contact maps for each stage showing the distance differences for formed (orange) or broken (purple) contacts are shown.

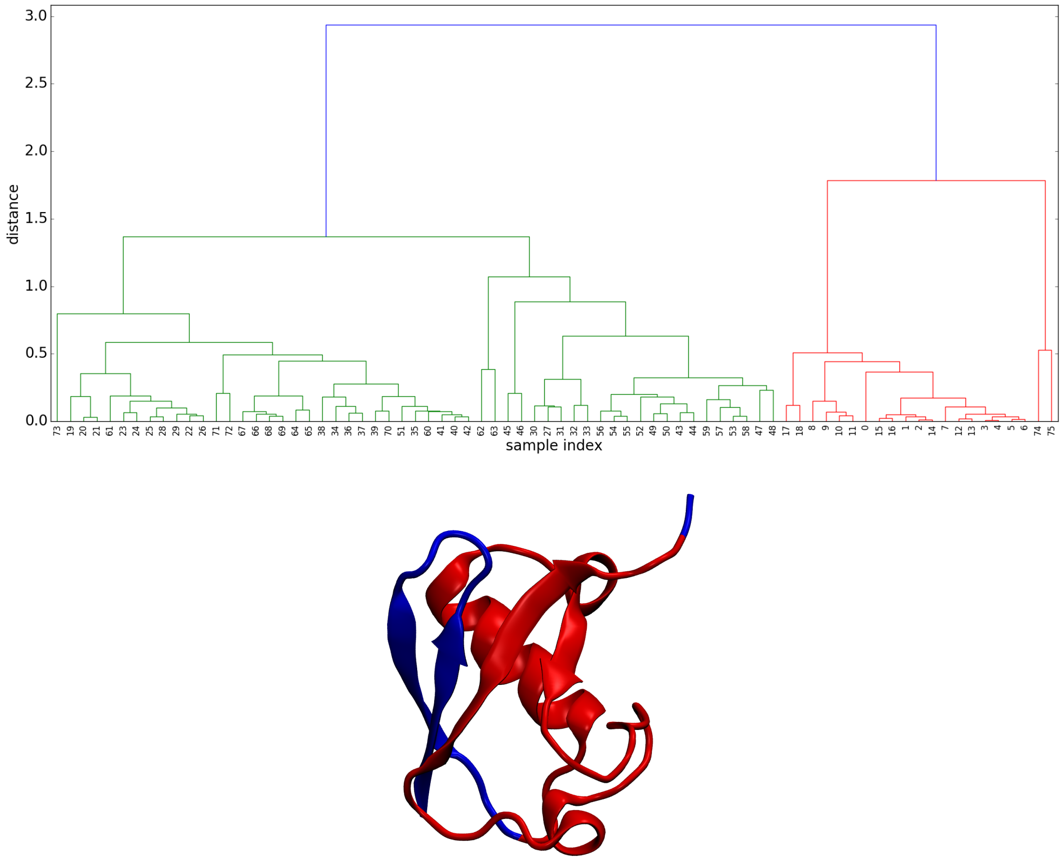

Figure S5: Top: The dendrogram of ubiquitin residues when clustering based on inter-residue cross-correlation. Bottom: The structure of ubiquitin colored by a two-cluster division based on the above dendrogram. The first 19 residues, forming the hairpin β_1-β_2, form one cluster along with the last two residues (74-75). This is because all of these residues had correlated contact losses in the first part of the trajectory. The internal 55 residues (19 to 73) unfolded later, therefore they belong to the other cluster.

4 Equilibrium simulation on α-synuclein fragment and E46K mutant

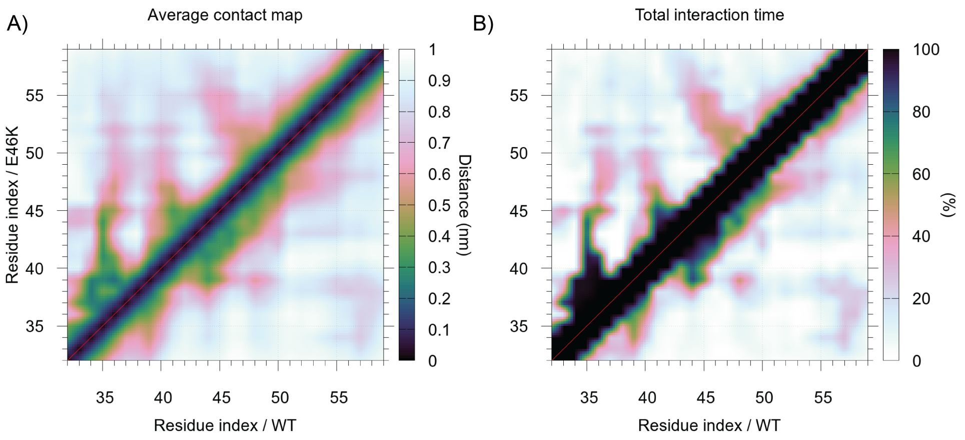

Figure S6: Conformational dynamics of a wild-type and E46K mutant of an α-synuclein fragment investigated through the analysis of contacts. (A) Asymmetric average contact map for the wild-type (lower triangle) and E46K mutant (upper triangle) of an α-synuclein molecule calculated by CONAN. (B) Total interaction time as calculated by CONAN for both the simulated systems (WT and E46K are shown in the lower triangles and upper triangles respectively).

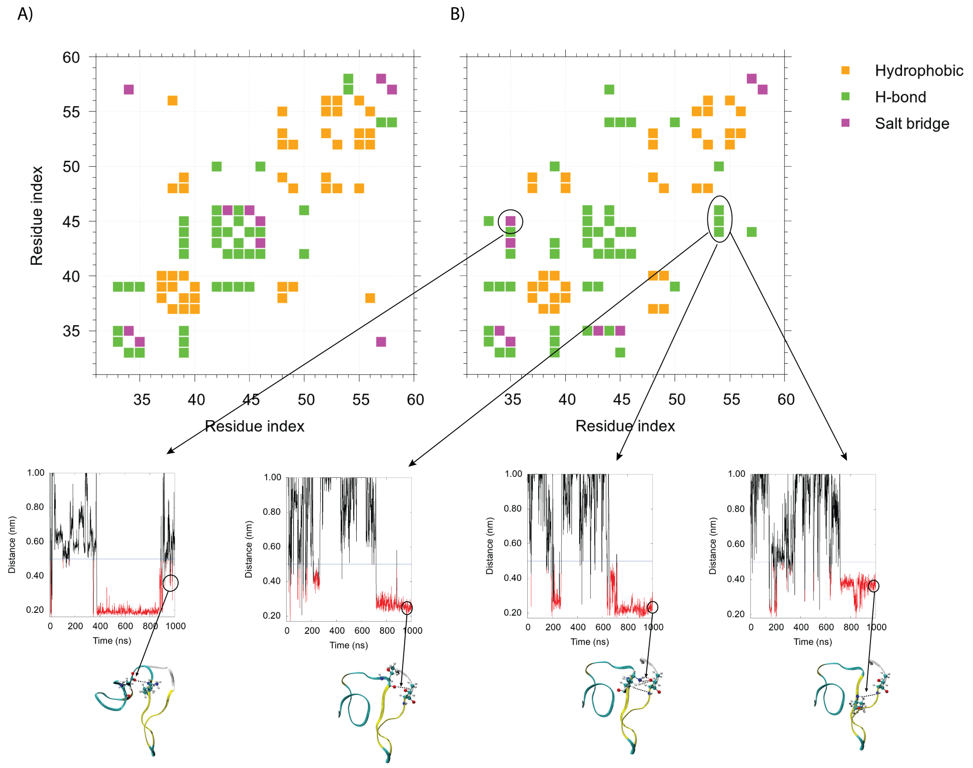

Figure S7: Classification of interaction types performed by CONAN. (A) and (B) show the contact maps colored according to the interaction types occurring within the simulated molecules for the WT (A) and E46K mutant (B) of the simulated α-synuclein molecules. The interaction network mainly responsible for the α to β conformational switch are circled and molecular distances as a function of simulated time are shown in the panel below and drawn in black or red when they are above or below the 0.5 nm cutoff respectively.

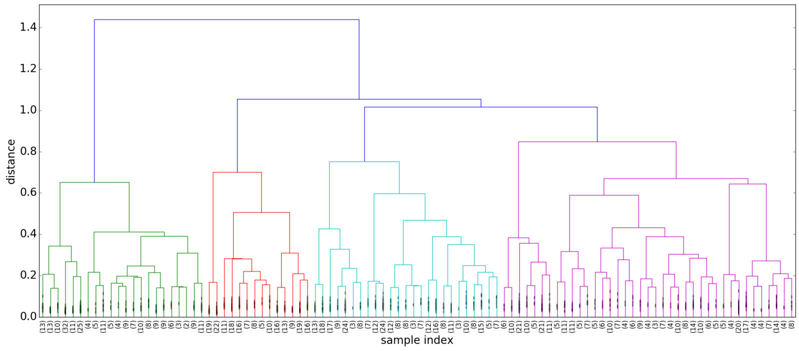

Figure S8: Dendrogram of the α-synuclein fragment (wild type) trajectory.

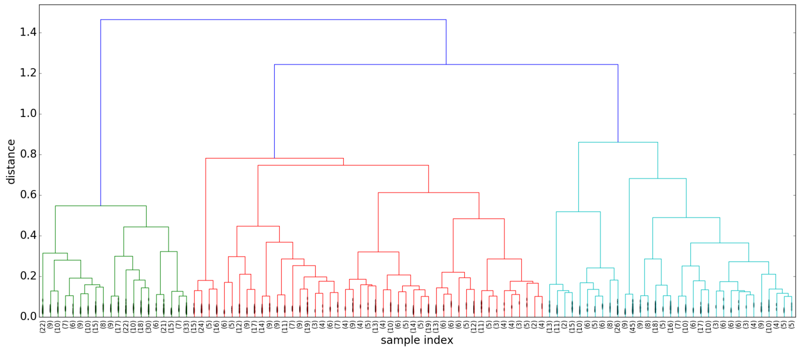

Figure S9: Dendrogram of the α-synuclein fragment (E46K mutant) trajectory.

5 Clustering non-equilibrium pathways: desmoplakin

CONAN can classify *pathways* rather than frames or residues. To this end, we will use the desmoplakin unfolding trajectories from Ref. [16]. This clustering could be done by linking the python executable as a library, but here, we will illustrate this doing the following method:

1. Run a separate CONAN run on each unfolding trajectory $n = 1..N_{\mathrm{traj}}$.
2. Define an inter-residue "distance" based on $d_{ij}(n) = 1 - t^{ij}_{\mathrm{lifetime}}$, where $t^{ij}_{\mathrm{lifetime}}$ is obtained from point 1.
3. Create a fictitious trajectory of N_{traj} frames, where this d_{ij} acts as a distance and n is a time.
4. Cluster this fictitious trajectory. The resulting classification will characterize the unfolding pathways.

Based on the obtained dendrogram (Fig. S10), we could reasonably choose two or three clusters. For simplicity, we choose two of them.

The average "contact maps" of the two clusters of trajectories are shown in Fig. S11. The main difference between the two clusters is the relatively earlier unfolding of helix 5C.

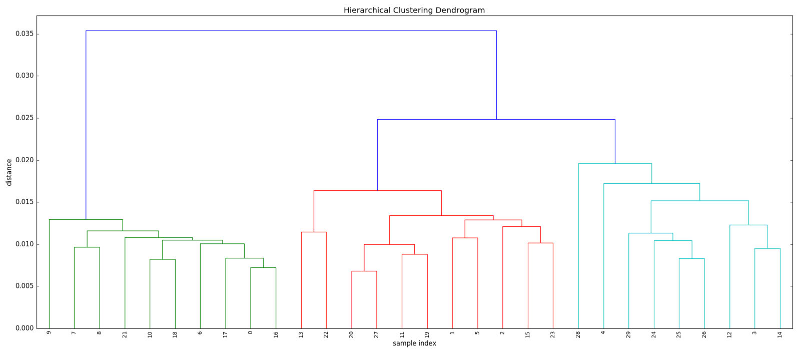

Figure S10: Classification of the desmoplakin unfolding trajectories from Daday et al, Sci Rep 2017. Indices from 0-9 refer to unfoldings at v=1.0 m/s, 10-19 to those at v=1/3 m/s, and 20-29 to those at v=1/10 m/s.

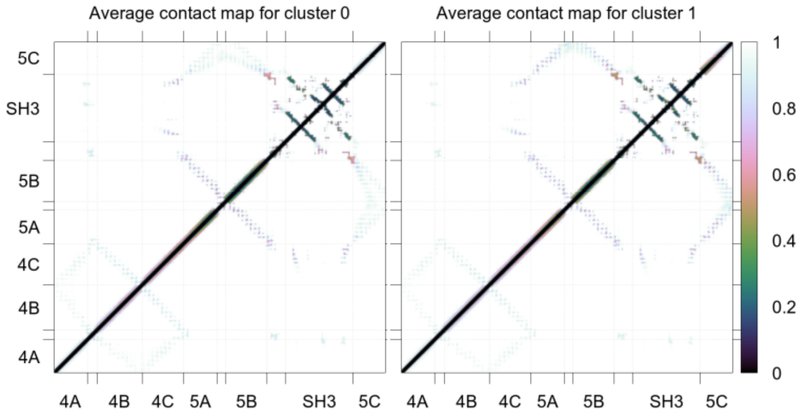

Figure S11: Characteristics of the two clusters of unfolding trajectories for desmoplakin. The shown quantity is 1-(interaction lifetime) computed on the unfolding trajectories.

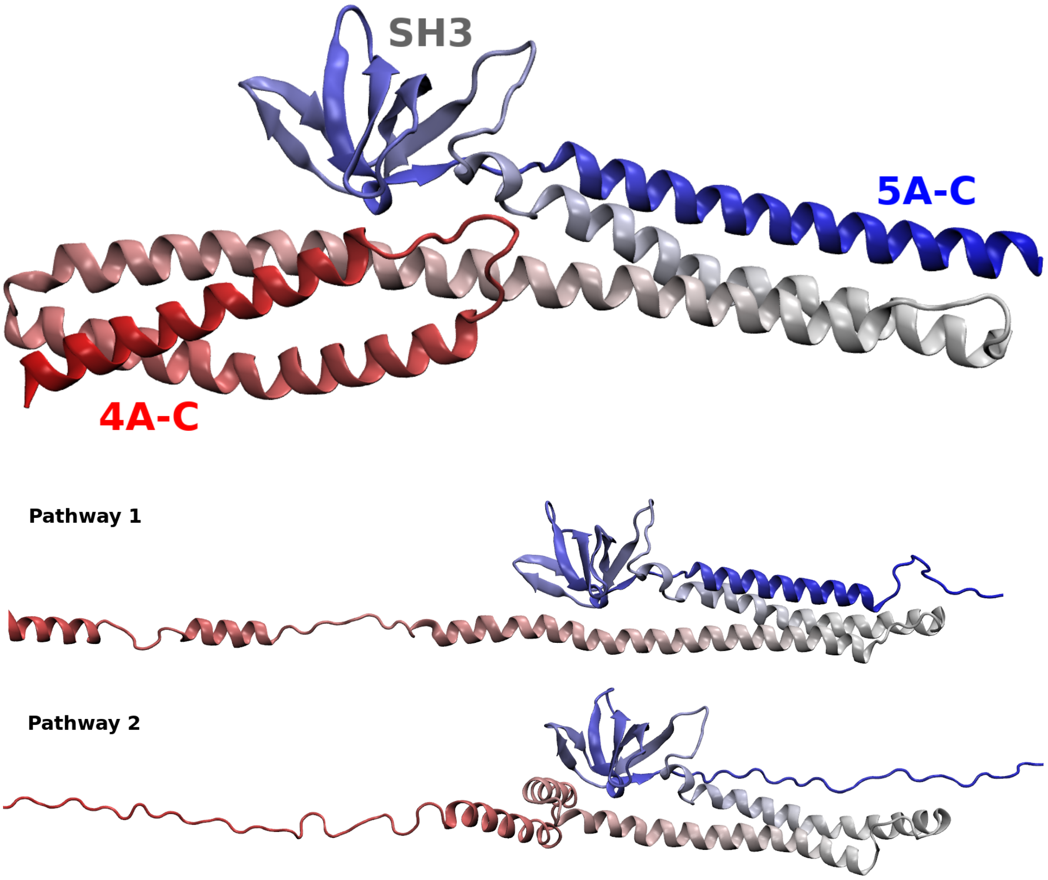

Figure S12: Top: Initial structure of spectrin repeats 4-5 of desmoplakin, consisting of helices 4A-4C, (red to gray) 5A-5C (gray to blue) and an SH3 domain insertion. Bottom: Partially unfolded states in the central two unfolding simulations of desmoplakin. Both correspond to a relative extension of about 20 nm. Helix 5C is fully unfolded in pathway 1 and almost fully intact in pathway 2.

6 Asymmetric contact maps: villin headpiece docking

We analyzed a sample trajectory containing 8 villin headpiece domains in a cubic box. This trajectory was kindly provided by Michael Feig's group, related to Ref. [10]. The trajectory is 2μs long, sampled every 100p̃s (20000 frames).

We show here CONAN's ability to analyze inter-chain interactions. There are 8 chains, so there are a total of 28 possible pairs of chains. We proceeded as follows:

1. We analyzed the trajectory using the 8 chains as "residues" and considering only heavy atom interactions. The 28 possible pairs are considered to interact with cutoffs of $r_{\mathrm{inter}} = 3$ Å to form and $r^{\mathrm{high}}_{\mathrm{inter}} = 5$ Åto break. An example time-evolution plot is in Figure S13.

2. Out of each of the 28 pairs, we extract interactions which last at least 10 ns. We obtain contact maps from these interactions once every 1 ns. This defined a total of 188 interactions and 8376 "binding poses". Each of the 28 pairs of chains formed at least 2 interactions during the simulations.

3. We perform a hierarchical clustering of these 8376 binding poses.

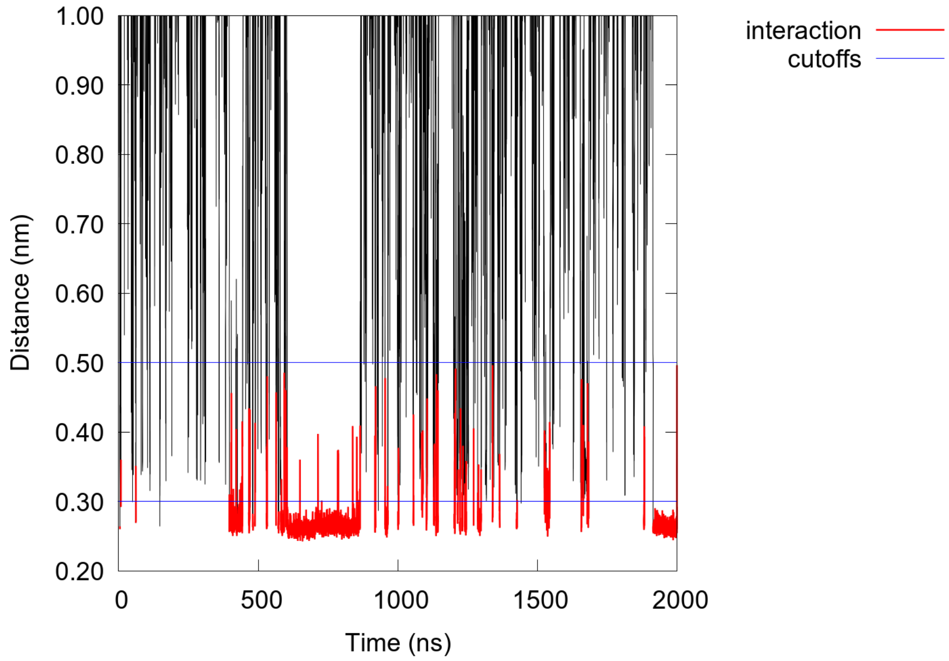

Figure S13: Inter-chain distance between two villin headpiece molecules. Interactions form at any inter-heavy atom distance 3Åand break at 5Å. Only one example plot is shown, the initial screening is based on 28 such inter-chain distance plots.

Based on the dendrogram in Fig. S14, we choose 3 clusters and analyze them further.

Cluster 0 contains 3099 frames, and the most characteristic interactions are salt bridges between R15 and D6 (23% between chains A-B and 15% between chains B-A). We show the overall lifetimes of interactions between the two chains as well as an example 3D snapshot (in which both copies of R15 are involved) in Fig. S15.

Cluster 1 contains 795 frames, and it only occurred once in the samples. Given that it lasted almost 800 ns, it represents an exceptionally strong docking pose. The example snapshot in Fig. S16 shows how one of the villin headpiece chains partially unfolds and the two chains form a strong hydrophobic packing.

Cluster 2 contains 4482 frames, and it is most well characterized by a hydrophobic packing between M1, L2 at the N-terminus of one chain and L35, F36 at the C-terminus (see Fig. S17) of the other. These interactions occurred about 15-20% of the frames in the clusters.

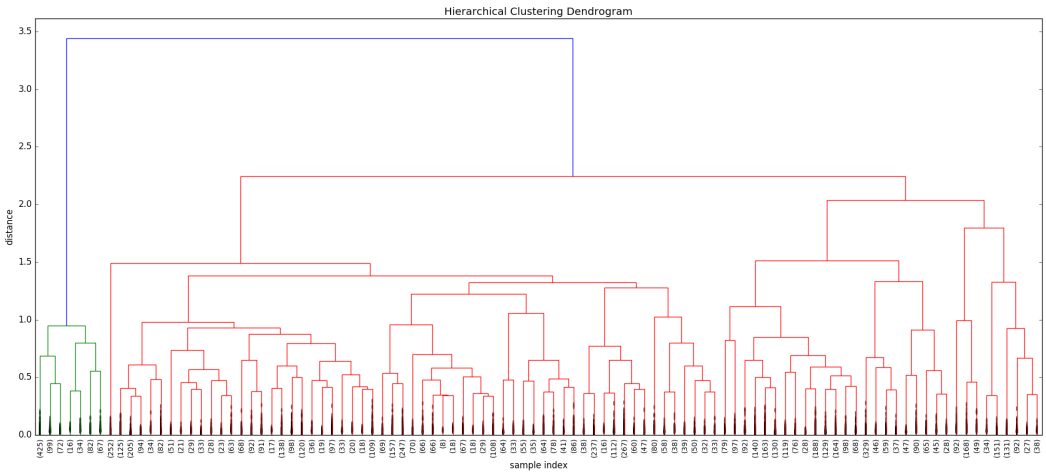

Figure S14: The top 100 nodes of the villin-villin docking dendrogram. Based on this dendrogram, we choose 3 clusters and analyze them in the following.

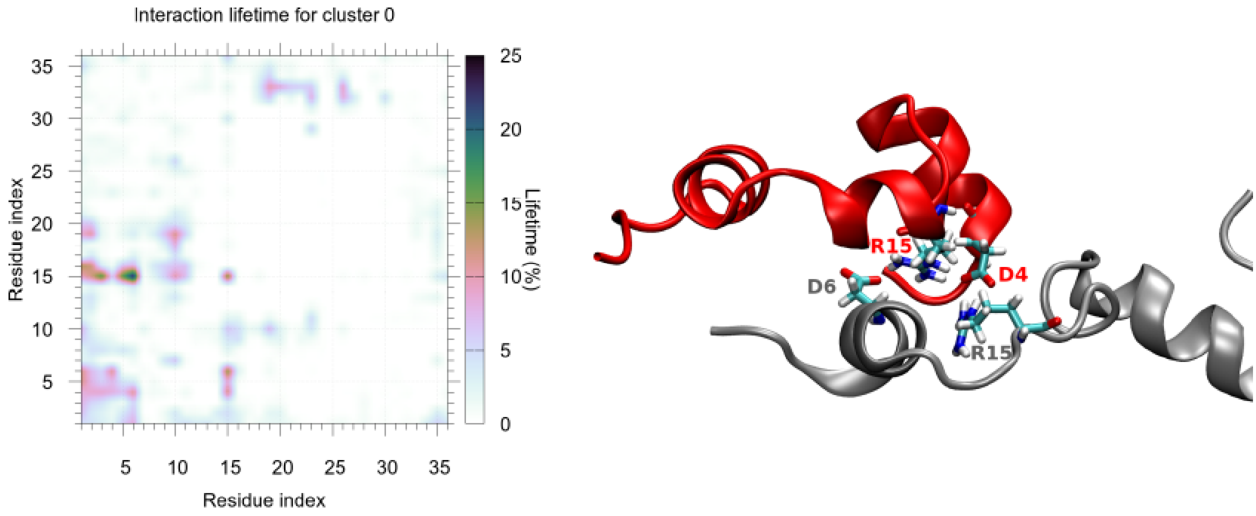

Figure S15: Cluster 0 of villin-villin docking poses. Left: inter-chain interaction lifetime characterizing the interaction. Right: snapshot illustrating the salt bridge R15-D6. In this case, R15 of the other chain also forms a salt bridge, with D4.

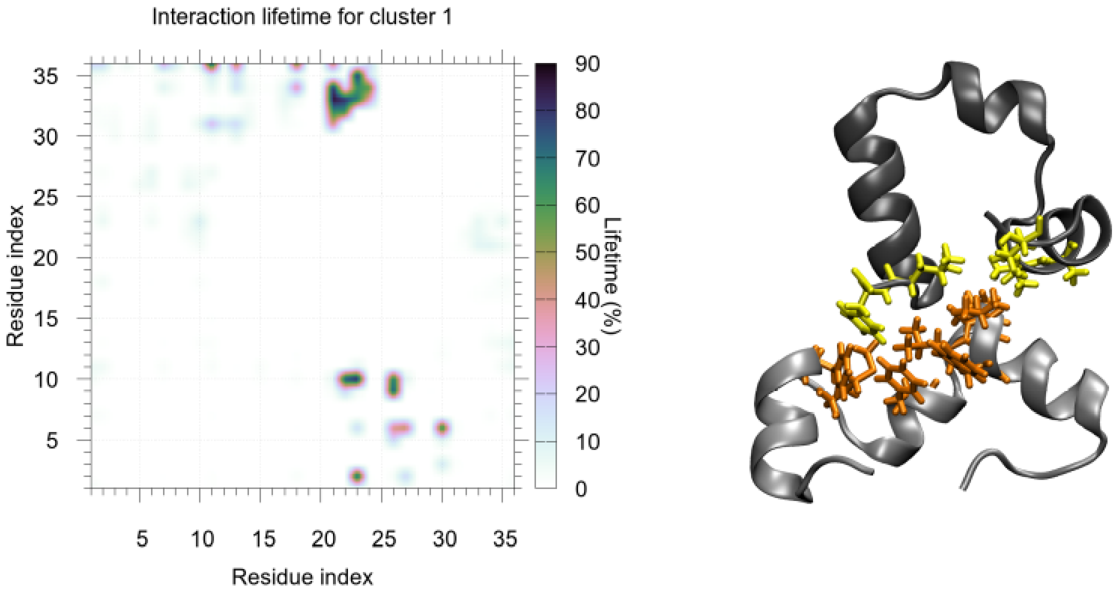

Figure S16: Cluster 1 of villin-villin docking poses. Left: inter-chain interaction lifetime characterizing the interaction. Right: snapshot illustrating the hydrophobic docking between the chains. All hydrophobic residues which are at most 5Å from the other chain are shown.

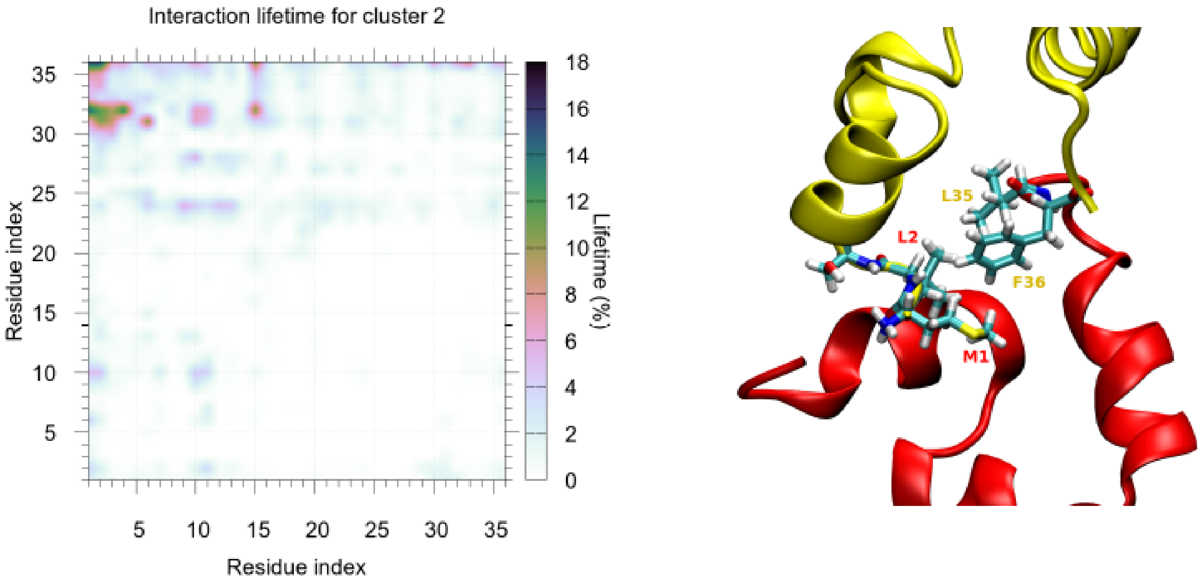

Figure S17: Cluster 2 of villin-villin docking poses. Left: inter-chain interaction lifetime characterizing the interaction. Right: snapshot illustrating the hydrophobic docking between the N-terminus of one chain and the C-terminus of the other.

7 All input files used in the writing of the main article

7.1 Ubiquitin equilibrium simulation

```
TRAJ md.xtc
COORD md.tpr
NLEVEL 1001
TRUNC  1.0
DT 100
TRUNC_INTER 0.5
GNUS_PATH ~/CONAN/input_gnuplotscripts/
DOMAINS domains.txt
PEARSON_TIME yes
PEARSON_OBS area_gyrate_100ps.txt
COORD_PDB ubi.pdb
ZOOM_LIST zoom_list.txt
```

7.2 Ubiquitin unfolding simulation

```
TRAJ pull.xtc
COORD pull.tpr
NLEVEL 1001
TRUNC  1.0
DT 100
TRUNC_INTER 0.5
GNUS_PATH ~/CONAN/input_gnuplotscripts/
K_RES_CLUSTERS 1-5
DOMAINS domains.txt
COORD_PDB ubi.pdb
```

7.3 α-synuclein fragment and E46K equilibrium simulations

```
TRAJ pull.xtc
COORD pull.tpr
NLEVEL 1001
NTERM 32
TRUNC  1.0
DT 100
TRUNC_INTER 0.5
K_TRAJ_CLUSTERS 2-5
GNUS_PATH ~/CONAN/input_gnuplotscripts/
```

7.4 Comparison between α-synuclein fragment wild type and E46K mutant

This is used by the secondary CONAN tool, conan.comp.py.

```
RUN_A ../WT
RUN_B ../E46K
TITLE_A "wildtype"
TITLE_B "E46K"
```

7.5 Desmoplakin clustering of lifetimes

For each of the 30 unfoldings, we used the following CONAN input:

```
TRAJ pull.xtc
COORD pull.tpr
NLEVEL 1001
NTERM 32
TRUNC  1.0
DT <deltat>
TRUNC_INTER 0.6
GNUS_PATH ~/CONAN/input_gnuplotscripts/
```

where deltat=0.25 nm/v (e.g., for $v = 1$ m/s, we take snapshots every 250 ps). This means that every pulling velocity contributes the same number of frames in the full contact analysis. Then, we created a file where we print $d_{ij} = 1 - t_{\mathrm{life}}$ (t_{life} is contained in the file lifetime .dat).

Then, using CONAN's REREAD feature, we read these 30 "frames" and cluster them:

```
REREAD .
TRUNC  1.0
K_TRAJ_CLUSTERS 2-3
GNUS_PATH ~/CONAN/input_gnuplotscripts/
```

7.6 Villin headpiece docking

We began by running CONAN on the entire trajectory, uniting all atoms from each protein as to have the same residue ID. We used this file (sys_chains .gro) as a structure file for the contact analysis. all .txt contains all 28 possible chain pairs.

```
TRAJ traj.gro
COORD sys_chains.gro
NLEVEL 1000
TRUNC 1.00
TRUNC_INTER 0.3
TRUNC_INTER_HIGH 0.5
GNUS_PATH ~/CONAN/input_gnuplotscripts/
RUN_MDMAT yes
ZOOM_LIST all.txt
```

The output of CONAN in the "zoomed in" version was next used to generate interesting fragments of the simulation time (any time at least 10 ns long where the interaction between the chains is uninterrupted), using simple awk commands. For each of these interesting time fragments, CONAN was used on the particular 72-residue fragment.

```
TRAJ traj.gro
COORD sys_chains.gro
NLEVEL 1000
TRUNC 1.00
INDEX index.ndx
TRUNC_INTER 0.5
TRUNC_INTER_HIGH 0.5
BEGIN <begin>
END <end>
GNUS_PATH ~/CONAN/input_gnuplotscripts/
RUN_MDMAT yes
ZOOM_LIST all.txt
```

7.7 Supplementary videos

- Supplementary Video V1: ubiquitin unfolding (framewise contact maps).
- Supplementary Video V2: ubiquitin unfolding (framewise differential contact maps w.r.t. initial frame).
- Supplementary Video V3: ubiquitin unfolding (framewise differential contact maps w.r.t. previous frame).
- Supplementary Video V4: α-synuclein fragment (wild type).
- Supplementary Video V5: α-synuclein fragment (E46K mutant).

References

[1] R. Ramage, J. Green, T. W. Muir, O. M. Ogunjobi, S. Love, and K. Shaw, "Synthetic, structural and biological studies of the ubiquitin system: the total chemical synthesis of ubiquitin.," *Biochem. J.*, vol. 299, pp. 151–158, 1994.

[2] E. A. Mirecka, H. Shaykhalishahi, A. Gauhar, Ş. Akgül, J. Lecher, D. Willbold, and M. Stoldt, "Sequestration of a β-hairpin for control of α-synuclein aggregation.," *Angewandte Chemie International Edition*, vol. 53, pp. 4227–4230, 2014.

[3] Y. Duan, C. Wu, S. Chowdhury, M. C. Lee, G. Xiong, W. Zhang, R. Yang, P. Cieplak, R. Luo, T. Lee, J. Caldwell, J. Wang, and P. Kollman, "A point-charge force field for molecular mechanics simulations of proteins based on condensed-phase quantum mechanical calculations.," *J. Comp. Chem.*, vol. 24, pp. 1999–2012, 2003.

[4] W. L. Jorgensen, J. Chandrasekhar, and J. D. Madura, "Comparison of simple potential functions for simulating liquid water.," *J. Chem. Phys.*, vol. 79, pp. 926–935, 1983.

[5] G. Bussi, D. Donadio, and M. Parrinello, "Canonical sampling through velocity rescaling," *J. Chem. Phys.*, vol. 126, p. 014101, 2007.

[6] M. Parrinello and A. Rahman, "Polymorphic transitions in single crystals: A new molecular dynamics method.," *J. Appl. Phys.*, vol. 52, pp. 7182–7190, 1981.

[7] D. Van Der Spoel, E. Lindahl, B. Hess, G. Groenhof, A. E. Mark, and H. J. C. Berendsen, "GROMACS: Fast, flexible, and free.," *J. Comp. Chem.*, vol. 26, pp. 1701–1718, 2005.

[8] W. Humphrey, A. Dalke, and K. Schulten, "VMD: Visual molecular dynamics," *J. Mol. Graph.*, vol. 14, pp. 33–38, 1996.

[9] J. E. Stone, "An efficient library for parallel ray tracing and animation," Master's thesis, 1998.

[10] G. Nawrocki, P. Wang, I. Yu, Y. Sugita, M. Feig, *et al.*, "Slow-down in diffusion in crowded protein solutions correlates with transient cluster formation," *J. Phys. Chem. B*, vol. 121, pp. 11072–11084, 2017.

[11] B. Brooks, C. Brooks, III, A. MacKerell, Jr, L. Nilsson, R. Petrella, B. Roux, Y. Won, G. Archontis, C. Bartels, S. Boresch, A. Caflisch, L. Caves, Q. Cui, A. Dinner, M. Feig, S. Fischer, J. Gao, M. Hodoscek, W. Im, K. Kuczera, T. Lazaridis, J. Ma, V. Ovchinnikov, E. Paci, R. W. Pastor, C. B. Post, J. Z. Pu, M. Schaefer, B. Tidor, R. M. Venable, H. L. Woodcock, X. Wu, W. Yang, D. York, and M. Karplus, "CHARMM: The biomolecular simulation program," *J. Comp. Chem.*, vol. 30, pp. 1545–1614, 2009.

[12] R. Best, W. Zheng, and J. Mittal, "Balanced protein-water interactions improve properties of disordered proteins and non-specific protein association," *J. Chem. Theory Comput.*, vol. 10, pp. 5113–5124, 2014.

[13] F. Sittel, J. Abhinav, and G. Stock, "Principal component analysis of molecular dynamics: On the use of cartesian vs. internal coordinates," *J. Chem. Phys*, vol. 141, p. 014111, 2014.

[14] H. Flyvbjerg and H. G. Petersen, "Error estimates on averages of correlated data," *J. Chem. Phys.*, vol. 91, p. 461, 1989.

[15] J. H. Ward, Jr., "Hierarchical grouping to optimize an objective function," *J. Am. Stat. Assoc.*, vol. 58, pp. 236–244, 1963.

[16] C. Daday, K. Kolšek, and F. Gräter, "The mechano-sensing role of the unique SH3 insertion in plakin domains revealed by molecular dynamics simulations," *Sci. Rep.*, vol. 7, p. 11669, 2017.
