# Supplementary material for: CONAN: A Tool to Decode Dynamical Information from Molecular Interaction Maps
Source: Biophys J. 2018 Mar 27;114(6):1267–73. doi: 10.1016/j.bpj.2018.01.033 (PMC5883949; doi:10.1016/j.bpj.2018.01.033)
Supplement: Document S2. Article plus Supporting Material [file mmc7.pdf]

Biophysical Journal
Computational Tool

Biophysical Society

CONAN: A Tool to Decode Dynamical Information from Molecular Interaction Maps

Davide Mercadante,[1] Frauke Gräter,[1,2] and Csaba Daday[1,2,*]
[1]Interdisciplinary Center for Scientific Computing, Heidelberg University, Mathematikon, Heidelberg, Germany and [2]Molecular Biomechanics Group, Heidelberg Institute for Theoretical Studies, Heidelberg, Germany

ABSTRACT The analysis of contacts is a powerful tool to understand biomolecular function in a series of contexts, from the investigation of dynamical behavior at equilibrium to the study of nonequilibrium dynamics in which the system moves between multiple states. We thus propose a tool called CONtact ANalysis (CONAN) that, from molecular dynamics (MD) trajectories, analyzes interresidue contacts, creates videos of time-resolved contact maps, and performs correlation, principal component, and cluster analysis, revealing how specific contacts relate to functionally relevant states sampled by MD. We present how CONAN can identify features describing the dynamics of ubiquitin both at equilibrium and during mechanical unfolding. Additionally, we show the analysis of MD trajectories of an α-synuclein mutant peptide that undergoes an α-β conformational transition that can be easily monitored using CONAN, which identifies the multiple states that the peptide explores along its conformational dynamics. The high versatility and ease of use of the software make CONAN a tool that can significantly facilitate the understanding of the complex dynamical behavior of proteins or other biomolecules. CONAN and its documentation are freely available for download on GitHub.

INTRODUCTION

Protein dynamics critically determine biological function. Given how complex and nonisotropic proteins are, describing their dynamics is a challenging task. Contact maps represent a formidable instrument for depicting intramolecular or intermolecular interactions. By definition, they are two-dimensional (2D) matrices that are built employing a user-defined cutoff on the basis of a chosen interresidue distance criterion relevant to the investigated molecular behavior (1). They have been used to predict and describe the formation of protein complexes (2), to reconstruct the three-dimensional (3D) structure of proteins (3), in protein folding studies (4), or, more recently, as a base of Markov-state models for protein folding (5).

Contact maps encode all the structural information (secondary and tertiary structure) characterizing the investigated molecule. Helices can be identified by a thickening of the matrix diagonal, whereas parallel and antiparallel β-strands are represented as narrow contact stretches orthogonal or parallel to the diagonal of the matrix, respectively, and similar patterns can show parallel and antiparallel packing in general. Thus, contact maps encode structural information through an exhaustive 2D representation of distances.

However, molecular simulations additionally sample conformational properties of molecules through time. The incorporation of dynamical information in contact matrices is extremely valuable to describe structural changes observed along a trajectory. At equilibrium, the analysis of contacts along MD trajectories can be useful to understand the lifetime of contacts across the structure, the achievement of convergence, or new conformational states explored by a system (6,7). Contact maps can also reveal information about the stability of intramolecular interactions during unfolding of the protein, as for example in force-probe molecular dynamics simulations (8). In these nonequilibrium conditions, the time-resolved analysis of contact maps can show the order in which contacts break along the pulling trajectory, which interactions are mostly responsible for withstanding the applied force, can identify unfolding intermediates, and whether there are multiple unfolding pathways.

Several tools have been created to visualize contact matrices and map the information they carry onto the 3D structure of molecules (3,9–12), but they mostly focus on encoding static (structural) information in them or require substantial input from the user. We here present a tool

Submitted November 20, 2017, and accepted for publication January 22, 2018.

*Correspondence: csaba.daday@h-its.org

Editor: D. Peter Tieleman.

https://doi.org/10.1016/j.bpj.2018.01.033

© 2018 Biophysical Society.

This is an open access article under the CC BY license (http://creativecommons.org/licenses/by/4.0/).

named CONtact ANalysis (CONAN) that, in addition to generating time-averaged quantities, also captures and encodes dynamical information from molecular dynamics (MD) trajectories into contact maps in a highly automated manner, requiring no scripting experience on the part of the user.

CONAN automatically generates publication-quality images and videos showing the evolution of contacts along MD trajectories and additionally provides a series of statistical analysis correlating the formation and rupture of contacts with time or with any observable of interest. It also implements contact map-based alternatives to commonly used techniques in computational biophysics, such as root-mean-square deviation (RMSD) and root-mean-square fluctuations (RMSFs), cluster analysis (in particular, hierarchical clustering (13)), and principal component analysis (PCA) (similar to (14)), thus offering the inherent advantage of avoiding the fitting procedure and/or the use of average coordinates required to perform these analyses based on atomic positions. CONAN also introduces an interresidue cross correlation based on the number of contacts rather than molecular fluctuations, again eschewing any interframe fitting.

We here report the analysis of test cases in which force-probe and equilibrium MD runs are performed on ubiquitin (15) and on a mutant peptide of α-synuclein (16), respectively. In addition, we illustrate how CONAN can also analyze protein-protein interactions by classifying villin headpiece homodimers (17) (Supporting Material). These test systems demonstrate the capabilities of CONAN in identifying conformational transitions. We show how the presented tool is helpful in the detailed description of an unfolding pathway of ubiquitin. The simulated α-synuclein fragment, on the other hand, undergoes an α to β transition that can be readily identified by CONAN. Contact maps colored by the chemical nature of the most long-lived interactions allow the straightforward identification of the chemical moieties playing a predominant role in the observed transition. Overall, the set of features comprised in CONAN make it a powerful tool for the analysis of MD simulations.

METHODS

We here restrict our discussion to the definition and choice of cutoffs. See Supporting Material and the online manual for a full explanation of possible outputs and methods.

Definition of contacts

CONAN can be customized with up to three different cutoff distances:

$\mathrm{r_{cut}}$ This is the main cutoff value. Any residue pair without any atoms within this cutoff is disregarded.

$\mathrm{r_{inter}}$ This is the cutoff value under which interactions are formed.

$\mathrm{r_{inter}^{high}}$ This is the cutoff value over which interactions are broken.

The main quantity CONAN uses is the interresidue distance defined as:

$$\mathrm{r_{ij}(t)} = \begin{cases} \mathrm{r_{ij}^{min}(t)} & \mathrm{r_{ij}^{min}} < \mathrm{r_{cut}} \\ \mathrm{r_{cut}} & \mathrm{r_{ij}^{min}} \geq \mathrm{r_{cut}}, \end{cases}$$

where $\mathrm{r_{ij}^{min}(t)}$ is the minimum distance between atoms from residues i and j. The user can choose a given class of atoms of interest (C_α, backbone atoms, side chains, any heavy atoms, any atom, etc.). In the analysis presented below, we always consider only heavy atom distances, as it reduces the search space by a factor of $\sim$4 without losing significant information, a main cutoff $\mathrm{r_{cut}} = 1$ nm, and interactions defined using the same cutoff, $\mathrm{r_{inter}} = \mathrm{r_{inter}^{high}} = 0.5$ nm. The approach of using two different cutoffs for breaking and forming interactions, proposed before by Best et al. (18), avoids the underestimation of the stability of interactions due to a few outlier frames. However, in this case, we are using only one value for simplicity.

Functionality of CONAN

CONAN has been designed with the purpose of being both user-friendly and versatile and requires few extra packages installed. To run the contact-based analysis of the performed MD simulations, the user provides a single input file with a set of keywords that the software interprets to perform the desired analyses. The input files used in the example cases used here are in the Supporting Material. To build contact maps, CONAN utilizes the mdmat utility provided in the GROMACS MD simulation engine (19) and interprets the output to retrieve the required statistics (Fig. 1). Using gmx mdmat has the inherent advantage of being compatible with other GROMACS tools (for example, using the custom index files). We stress, however, that a series of tools are available to convert trajectories to GROMACS format, and therefore CONAN is also compatible with results obtained from other packages. In the Supporting Material, we illustrate this by analyzing a trajectory obtained from CHARMM (Accelrys, San Diego, CA).

CONAN is programmed in Python 3 and is therefore easily available as a package as well. Given the possibly costly memory requirements of $\propto N_{\mathrm{res}}^2 \times N_{\mathrm{frames}}$ (number of residues squared times number of frames), we took particular care of economical data storage. All of the memory-sensitive quantities are stored in a sparse way and using half-precision (numpy's float16 structure) for efficient memory use. Furthermore, CONAN can be run in an "economy mode" in which only one frame and only aggregate quantities are stored, thereby reducing the memory usage to a minimum. Analogously, since the plain-text matrices can occupy significant disk space, the user also has an option to only store aggregate ones and/or only the .png files. Further information on requirements can be found in the manual. The current main bottleneck is generating the framewise .png files in gnuplot, and the CONAN-specific commands are executed very efficiently through vectorized instructions implemented in numpy and scipy. For example, turning off .png generation, the given example of ubiquitin unfolding, involving 1976 pairs and 2357 frames, requires $\sim$33 s on a single core of an Intel i7-4770 (Intel, Santa Clara, CA) central processing unit for a standard analysis, and 84 s for a full analysis (correlations, both types of cluster analysis, and PCA); in fact, even these timings are mostly input/output-limited.

CONAN uses gnuplot (20) to plot at each time step the interaction maps calculated from the contact matrix and collates them using video encoding (×264 in this case) for the creation of videos that show time-resolved evolution of the contacts. As color schemes for the creation of contact maps, we use cubehelix (21) for distances and times (sequential data) and an 11-class version of Cynthia Brewer's PuOr (purple-orange) scheme (22) for divergent data (changes or correlation coefficients), in which we set the central point to be exactly white (#FFFFFF in hexadecimal). CONAN, its documentation, and example use cases are freely available for download at https://github.com/HITS-MBM/conan under

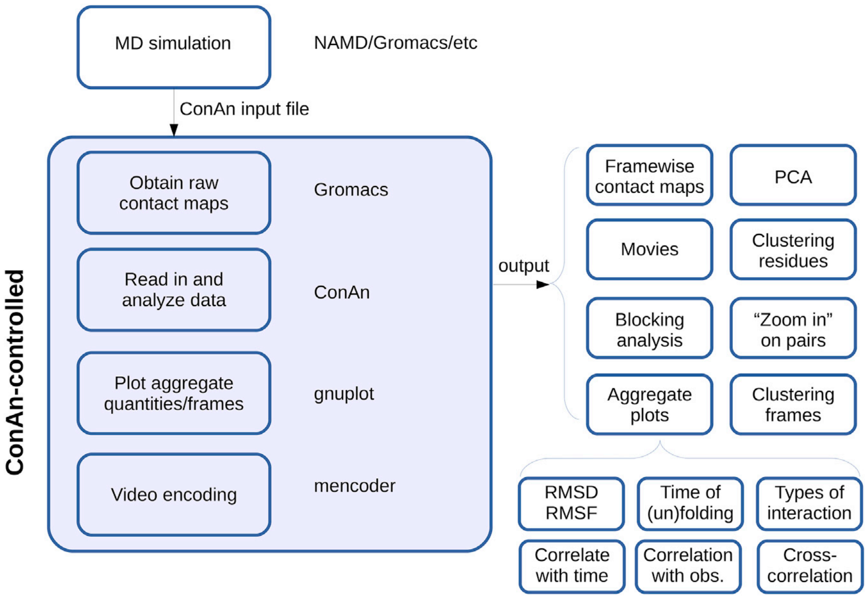

FIGURE 1 Flowchart illustrating CONAN's main workflow. The flowchart shows the different tools (left-hand side of the figure) used by CONAN to output a series of analysis required by the user (right-hand side of the figure). The kinds of analysis to perform, as well as the corresponding parameters, are defined in the CONAN input file. All of the plots except this figure and Fig. 4 *A* are based on standard outputs from CONAN, augmented mainly only by 3D structures and highlights. obs., observation. To see this figure in color, go online.

GPL 3.0. Further examples and explanations can be found on http://contactmaps.blogspot.com.

RESULTS AND DISCUSSION

Ubiquitin equilibrium dynamics analyzed with CONAN

Ubiquitin is a small molecule structurally characterized by different secondary structure elements (one α-helix and a four strands β-sheet), and a useful model system for computational and experimental studies investigating protein structural and dynamical behavior: from the investigation of protein folding (23) to the testing of protein force fields used in MD simulations (24). We have herein chosen this molecule to provide a test case representing the capabilities of CONAN and therefore have performed equilibrium and force-probe MD simulations to illustrate how contact map analysis, as provided by CONAN, can lead to a deep understanding of protein functional dynamics.

At equilibrium, we computed the average contact map of the protein (Fig. 2 *C*), which clearly shows that the protein's secondary structure is stable, with the α_1 helix, the antiparallel packing between strands β_1-β_2, β_3-β_4, and the parallel packing between strands β_2-β_3 and between β_1-β_4. Furthermore, we can also observe fluctuations between interresidue distances (Fig. 2 *D*), which are dominated by the motion of the C-terminal linker with respect to the β_3 strand and its immediate vicinity. RMSFs (Fig. 2 *B*) are a widely used observable to monitor, on average, the dynamics of residues within a structure. CONAN's interresidue distance fluctuations can be considered as more reliable than RMSF values, especially when large fluctuations occur, as average positions can be physically meaningless. Furthermore, the output contains the most relevant interactions (changing only within values lower than the main cutoff) rather than identifying single residues.

The correlation of interresidue contacts with time shows a significant conformational rearrangement of the protein (Fig. 3 *A*). In particular, residue K48 drifts and increases its distance from residues I42 and R44 and approaches residue D58 with which it establishes a stable salt bridge. The salt bridge network newly formed between D51, R54, D58, and K48 is responsible for this stable conformation observed at the end of the simulated trajectory and representing a stable conformational state of the protein (Fig. 3). The correlation map clearly pinpoints the relevant regions in the 3D overlap of structures (Fig. 3 *A*). The conformational rearrangement is also captured in the interresidue cross correlation, which identifies a competitive behavior between R42 and D58 (see Fig. S1 and further explanation there).

This kind of analysis can be useful to diagnose drifts from the starting structure (for example, a crystal structure or a homology model), which might also reflect a lack of convergence in the simulation: in the (usually not achievable) limit of full exploration of the phase space, none of the interresidue or interdomain distances would show a significant correlation with time. In this case, a relatively short simulation time (50 ns) has been chosen, as can be seen by the significant time correlations identified. CONAN interestingly resolves a "local drift," with some regions equilibrated and others still changing conformation; this would be quite difficult to define in terms of 3D coordinates.

As mentioned in the Methods, CONAN can also identify the sequence determinants of macroscopic quantities; we

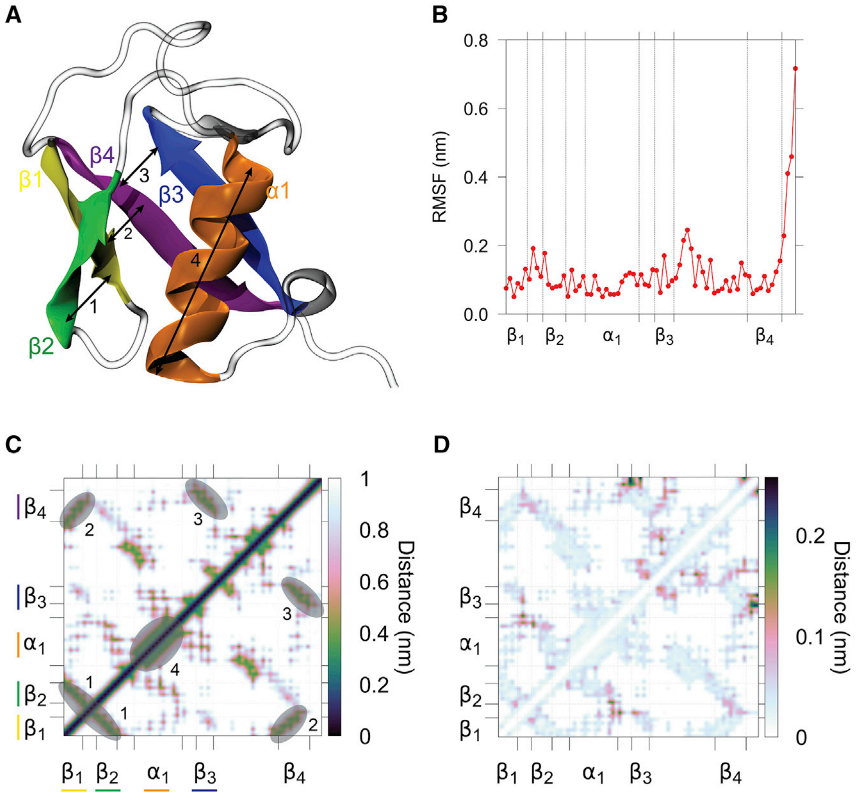

FIGURE 2 Dynamics of ubiquitin at equilibrium investigated by contact maps. (*A*) The structure of a ubiquitin molecule in a cartoon representation and colored according to its secondary structure elements and numbers denoting the most important groups of contacts are shown. (*B*) 3D coordinates-based root mean-square fluctuations (RMSFs) of ubiquitin are shown. (*C*) The average contact map is shown, with circles highlighting the most important contacts. (*D*) A symmetric contact map showing the standard deviation of contact distances along the trajectory. To see this figure in color, go online.

show how certain loop rearrangements explain changes in radius of gyration R_g in Fig. S2.

Unfolding trajectory of ubiquitin

Beyond studying the behavior of molecules at equilibrium, nonequilibrium trajectories can also be analyzed by looking at the formation or rupture of contacts. In this case, we show how the unfolding of ubiquitin, triggered by the application of an external force, can be mapped onto the protein's structure through the use of CONAN.

Fig. 4 *B* reports the last encounter of residue pairs (i.e., unfolding times) and shows how these events are clearly clustered around two moments. First, around 40 ns (*faint gray*),

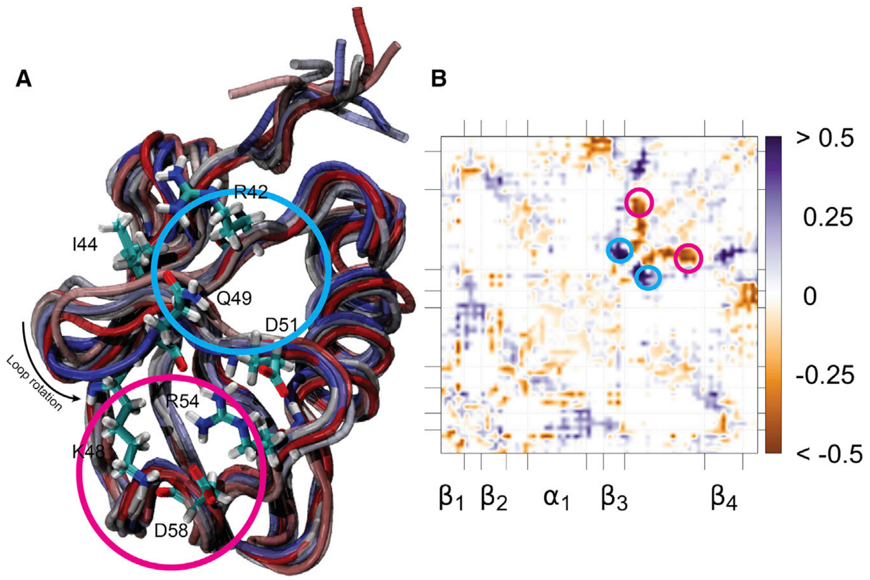

FIGURE 3 Correlation of interresidue distance with time. (*A*) An ensemble of ubiquitin at the equilibrium is shown. The conformers shown have been collected at every 5 ns and are colored as a function of time from red (start of the simulation) to blue (end of the simulation). The residues involved in pairs showing the highest correlations with time in map (*B*) are shown in licorice. (*B*) Correlation of interresidue distances with time are shown. Negative Pearson coefficients (*orange*) correspond to contact formation and positive ones (*purple*) correspond to contact ruptures. We highlight the largest changes in blue (ruptures) and pink (formations) in both plots.

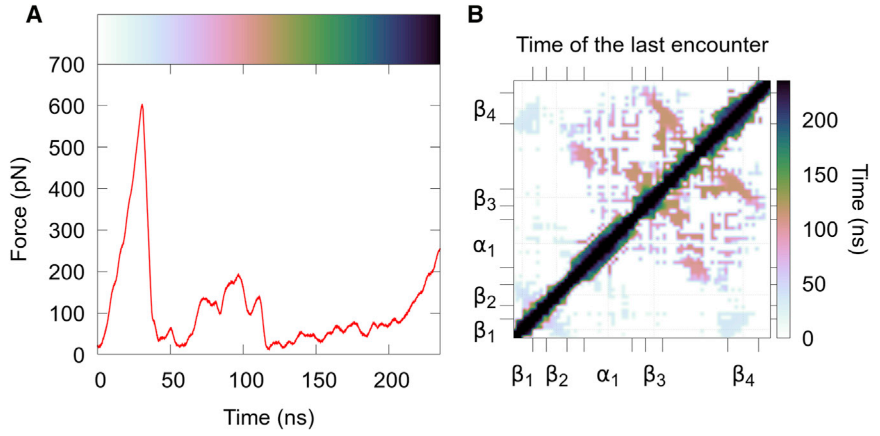

FIGURE 4 Force-driven unfolding of ubiquitin. (*A*) A force profile as a function of time is shown. (*B*) A time-encoded contact map showing the times of contact breaking is shown. The color code of subplot (*B*) is repeated in (*A*) for ease of comparison. To see this figure in color, go online.

β_1 loses contact with β_4 and β_2. Then, around 100 ns (*pink-orange*), α_1 and $\beta_3 - \beta_4$ lose contact with each other. Both of these events coincide with a drop in force (at $\sim$ 40 and $\sim$ 100 ns), reflecting barrier crossing events (Fig. 4 *A*). Finally, at various points in time after $\sim$150 ns (*green or darker*), α_1 and various small helical regions lose contact as the peptide becomes straighter. This last part of the unfolding happens without any resistance; therefore, the events happen without any cooperativity. The above-described pathway is in close agreement with previous MD studies of ubiquitin under force (25). Fig. S4 contains the differential contact maps of the four stages. Finally, the different domains of the ubiquitin unfolding can also be detected by residue clustering, clearly distinguishing the two β-strands from the other residues (see Fig. S5).

In short, Fig. 4 *B* summarizes an entire trajectory in a simple, intuitive 2D image. This object can further be used to cluster different independent unfolding trajectories either visually or through rigorous methods (see Supporting Material for an example of this).

α-Helix to β-strand transition of a mutant peptide from α-synuclein revealed by contact analysis

α-Synuclein is a well-studied disordered protein associated with the occurrence of Parkinson's disease (26–28). As in the case of other neurodegenerative disorders mediated by peptides, the molecular basis of the disease is related to a profound structural transition from an α-helix to a β-sheet, which is an aggregation-prone state (29,30). The mutation of glutamate to lysine at position 46 along the sequence (E46K) accelerates cytotoxicity of α-synuclein inside cells (31–34). We have chosen this system to show that CONAN can readily identify the pathological conformational transition from α-helix to β-strand. The simulations, performed in replicates of 1 μs, repeatedly show an abrupt conformational change from a starting α-helical conformation into a random coil and finally into a β-hairpin.

The cluster analysis on the trajectories of the simulated α-synuclein fragments reveals different behaviors for the wild-type and the E46K mutant. In the case of the wild-type, the fragment oscillates between three states defined by clusters 0, 1, and 3, which are mostly α-helical, while cluster 2 is scarcely populated and features a shorter helix and a more molten-globule configuration. The E46K mutant, on the other hand, shows a time-resolved evolution of the states leading to the formation of a β-hairpin, which is characteristic of intermediates assembling in fibrillary supramolecular structures (Fig. 5). We pinpoint the specific interactions responsible for these differences using CONAN (Figs. S6 and S7). The dendrograms of the wild-type and the mutant fragment can be found in Figs. S8 and S9.

CONCLUSIONS

Biological function is ultimately linked to molecular structure and its motions. The analysis of molecular contacts is exceptionally useful to map particular dynamic events that characterize the physiological or pathological behavior of macromolecules onto a molecular structure. Although bearing a plentiful amount of information and being extensively used to visualize static information (crystal structures or averages over the simulated time), contact maps have not been widely employed to visualize or interpret dynamical information. Here, we developed a tool that constructs time-resolved contact maps and also performs statistical, principal component, or cluster analyses to extract evidence of how specific contacts contribute to the overall observed dynamics of a macromolecule. CONAN is a tool developed with the purpose to combine the analysis of contacts to a series of analytic frameworks and to provide a highly automated instrument for a varied analysis of contacts with respect to the conformational ensembles explored by MD simulations. With the use of a single input file, CONAN computes contact maps and provides a range of publication-grade figures and videos that are automatically generated and show how specific contacts are responsible for an investigated behavior.

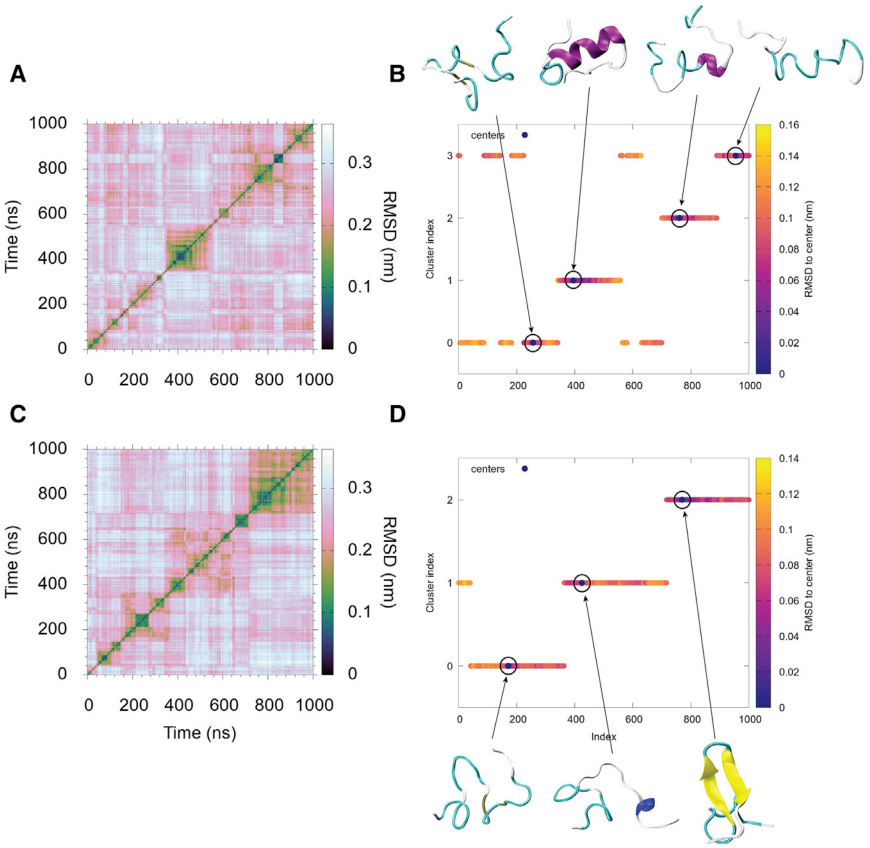

FIGURE 5 Contact-based cluster analysis of the wild-type and mutated (E46K) form of α-synuclein investigated using CONAN. (*A*) and (*C*) show the inter-frame RMSD of the trajectories (see Methods for details) describing the conformational dynamics of the simulated peptides. (*B*) and (*D*) show the clustering obtained for the simulated ensemble. The graphs describe the time evolution of clusters with points colored according to the distance of each conformation from the cluster medoid (*circled*). The 3D structures for each medoid color-coded by secondary structure are shown on the right-hand side of the figure. To see this figure in color, go online.

Interestingly, the analysis of unfolding trajectories by means of monitoring formation and rupture of contacts can not only reveal contact rupture but also the formation of new and variably transient contacts. Such analyses can be applied to understand folding of molecules in which exhaustive sampling is provided and can, for instance, reveal the formation of local element structures along a folding pathway (24,25) to reveal cooperative effects in protein-coupled binding and folding processes (35) or critically functional allosteric and catalytic events along protein structures (36). We believe that the highly automated output creation and the simplicity of the user interface is key to a widespread use of the contact-based analyses presented, which can enhance the understanding of molecular systems far beyond the test cases presented here.

SUPPORTING MATERIAL

Supporting Materials and Methods, seventeen figures, and five movies are available at http://www.biophysj.org/biophysj/supplemental/S0006-3495(18)30193-0.

AUTHOR CONTRIBUTIONS

D.M. and C.D. performed the simulations and data analysis. All three authors planned and wrote the manuscript. C.D. conceived of and wrote the software.

ACKNOWLEDGMENTS

All three authors acknowledge funding from the Klaus Tschira Foundation. We thank Michael Feig for providing MD data as a test case (Supporting Material). C.D. acknowledges help from Vedran Miletić for coding advice and help with setting up the GitHub repository and thanks Helene H. Thygesen for helpful discussions on statistical analysis.

REFERENCES

1. Mirny, L., and E. Domany. 1996. Protein fold recognition and dynamics in the space of contact maps. *Proteins.* 26:391–410.
2. Pulim, V., B. Berger, and J. Bienkowska. 2008. Optimal contact map alignment of protein-protein interfaces. *Bioinformatics.* 24:2324–2328.
3. Vassura, M., L. Margara, …, R. Casadio. 2008. FT-COMAR: fault tolerant three-dimensional structure reconstruction from protein contact maps. *Bioinformatics.* 24:1313–1315.
4. Vendruscolo, M., and E. Domany. 2000. Protein folding using contact maps. *Vitam. Horm.* 58:171–212.
5. Kellogg, E. H., O. F. Lange, and D. Baker. 2012. Evaluation and optimization of discrete state models of protein folding. *J. Phys. Chem. B.* 116:11405–11413.
6. Nath, A., M. Sammalkorpi, …, E. Rhoades. 2012. The conformational ensembles of α-synuclein and tau: combining single-molecule FRET and simulations. *Biophys. J.* 103:1940–1949.
7. Sethi, A., J. Tian, …, S. Gnanakaran. 2012. Identification of minimally interacting modules in an intrinsically disordered protein. *Biophys. J.* 103:748–757.
8. Grubmüller, H. 2005. Force probe molecular dynamics simulations. *Methods Mol. Biol.* 305:493–515.

9. Kozma, D., I. Simon, and G. E. Tusnády. 2012. CMWeb: an interactive on-line tool for analysing residue-residue contacts and contact prediction methods. *Nucleic Acids Res.* 40:W329–W333.

10. Rafferty, B., Z. C. Flohr, and A. Martini. 2014. Protein contact maps. https://nanohub.org/resources/contactmaps.

11. Vehlow, C., H. Stehr, …, M. Lappe. 2011. CMView: interactive contact map visualization and analysis. *Bioinformatics.* 27:1573–1574.

12. Krishnamani, V. 2016. CMPyMOL: a tool for protein contact-map analysis. *bioRxiv.* https://doi.org/10.1101/084269.

13. Ward, J. H., Jr. 1963. Hierarchical grouping to optimize an objective function. *J. Am. Stat. Assoc.* 58:236–244.

14. Sittel, F., A. Jain, and G. Stock. 2014. Principal component analysis of molecular dynamics: on the use of Cartesian vs. internal coordinates. *J. Chem. Phys.* 141:014111.

15. Ramage, R., J. Green, …, K. Shaw. 1994. Synthetic, structural and biological studies of the ubiquitin system: the total chemical synthesis of ubiquitin. *Biochem. J.* 299:151–158.

16. Mirecka, E. A., H. Shaykhalishahi, …, W. Hoyer. 2014. Sequestration of a β-hairpin for control of α-synuclein aggregation. *Angew. Chem. Int. Ed. Engl.* 53:4227–4230.

17. Nawrocki, G., P. H. Wang, …, M. Feig. 2017. Slow-down in diffusion in crowded protein solutions correlates with transient cluster formation. *J. Phys. Chem. B.* 121:11072–11084.

18. Best, R. B., and G. Hummer. 2010. Coordinate-dependent diffusion in protein folding. *Proc. Natl. Acad. Sci. USA.* 107:1088–1093.

19. Abraham, M. J., T. Murtola, …, E. Lindahl. 2015. Gromacs: high performance molecular simulations through multi-level parallelism from laptops to supercomputers. *SoftwareX.* 1:19–25.

20. Williams, T., and C. Kelley. 2013. Gnuplot 4.6: an interactive plotting program. http://gnuplot.sourceforge.net/.

21. Green, D. A. 2011. A colour scheme for the display of astronomical intensity images. *Bull. Astron. Soc. India.* 39:289–295.

22. Harrower, M., and C. A. Brewer. 2003. ColorBrewer.org: an online tool for selecting colour schemes for maps. *Cartogr. J.* 40:27–37.

23. Piana, S., K. Lindorff-Larsen, and D. E. Shaw. 2013. Atomic-level description of ubiquitin folding. *Proc. Natl. Acad. Sci. USA.* 110:5915–5920.

24. Lindorff-Larsen, K., P. Maragakis, …, D. E. Shaw. 2012. Systematic validation of protein force fields against experimental data. *PLoS One.* 7:e32131.

25. Das, A., and C. Mukhopadhyay. 2009. Mechanical unfolding pathway and origin of mechanical stability of proteins of ubiquitin family: an investigation by steered molecular dynamics simulation. *Proteins.* 75:1024–1034.

26. Flagmeier, P., G. Meisl, …, C. Galvagnion. 2016. Mutations associated with familial Parkinson's disease alter the initiation and amplification steps of α-synuclein aggregation. *Proc. Natl. Acad. Sci. USA.* 113: 10328–10333.

27. Li, W., N. West, …, M. K. Lee. 2005. Aggregation promoting C-terminal truncation of α-synuclein is a normal cellular process and is enhanced by the familial Parkinson's disease-linked mutations. *Proc. Natl. Acad. Sci. USA.* 102:2162–2167.

28. Volpicelli-Daley, L. A., K. C. Luk, …, V. M. Lee. 2011. Exogenous α-synuclein fibrils induce Lewy body pathology leading to synaptic dysfunction and neuron death. *Neuron.* 72:57–71.

29. Lashuel, H. A., C. R. Overk, …, E. Masliah. 2013. The many faces of α-synuclein: from structure and toxicity to therapeutic target. *Nat. Rev. Neurosci.* 14:38–48.

30. Narkiewicz, J., G. Giachin, and G. Legname. 2014. In vitro aggregation assays for the characterization of α-synuclein prion-like properties. *Prion.* 8:19–32.

31. Fredenburg, R. A., C. Rospigliosi, …, P. T. Lansbury, Jr. 2007. The impact of the E46K mutation on the properties of α-synuclein in its monomeric and oligomeric states. *Biochemistry.* 46:7107–7118.

32. Wise-Scira, O., A. Dunn, …, O. Coskuner. 2013. Structures of the E46K mutant-type α-synuclein protein and impact of E46K mutation on the structures of the wild-type α-synuclein protein. *ACS Chem. Neurosci.* 4:498–508.

33. Emmer, K. L., E. A. Waxman, …, B. I. Giasson. 2011. E46K human alpha-synuclein transgenic mice develop Lewy-like and tau pathology associated with age-dependent, detrimental motor impairment. *J. Biol. Chem.* 286:35104–35118.

34. Zarranz, J. J., J. Alegre, …, J. G. de Yebenes. 2004. The new mutation, E46K, of alpha-synuclein causes Parkinson and Lewy body dementia. *Ann. Neurol.* 55:164–173.

35. Kovacs, D., B. Szabo, …, P. Tompa. 2013. Intrinsically disordered proteins undergo and assist folding transitions in the proteome. *Arch. Biochem. Biophys.* 531:80–89.

36. Doshi, U., M. J. Holliday, …, D. Hamelberg. 2016. Dynamical network of residue-residue contacts reveals coupled allosteric effects in recognition, catalysis, and mutation. *Proc. Natl. Acad. Sci. USA.* 113: 4735–4740.

Biophysical Journal, Volume *114*

Supplemental Information

CONAN: A Tool to Decode Dynamical Information from Molecular Interaction Maps

Davide Mercadante, Frauke Gräter, and Csaba Daday

Supporting Information for:
CONAN: a tool to decode dynamical information from molecular interaction maps

D. Mercadante and F. Gräter and C. Daday

January 19, 2018

Abstract

1 Methods

1.1 Molecular dynamics simulations

A molecule of ubiquitin [1] and a fragment of α-synuclein extracted from a complex with an engineered protein [2] (PDB codes: 1UBI and 4BXL respectively) were placed in the center of dodecahedron boxes having volumes so that the shortest distance between periodic images was at least 3 nm. Hydrogen atoms were added and a topology was created by assigning to each particle parameters from the AMBER03 force field [3]. After topology creation, the proteins were energy minimized for 20000 steps using a steepest descent algorithm with a force tolerance of 20 kJ mol^{-1}nm^{-1} and a step size of 0.001. The system was then solvated with TIP3P water molecules [4]. Sodium (Na^+) and chloride (Cl^-) ions were added in order to reach an ionic strength of 0.15 M. After solvation and insertion of ions, the systems were firstly equilibrated in the NVT ensemble in which particles' velocities were generated according to a Boltzmann distribution obtained at 300 K. In the NVT step, which was carried out for 500 ps, the temperature was kept constant at 300 K using a V-rescale thermostat [5]. Subsequently to the NVT step, equilibration was continued in the NpT ensemble for additional 500 ps, through which temperature and pressure were kept constant at the values of 300 K and 1 bar respectively. As for the equilibration in NVT ensemble, temperature was kept constant by using a V- rescale thermostat with a time constant of 0.5 ps, whereas pressure was coupled through a Parrinello-Rahman barostat [6] in all dimensions (isotropic pressure coupling) with a time constant of 2.0 ps and a compressibility of $4.5 \cdot 10^{-5}$ bar^{-1}. During both NVT and NpT steps, a restraint on the positions of all the atoms of the proteins was applied using an external harmonic potential of 1000 kJ mol^{-1}nm^{-1}.

Production runs were then carried out in the NpT ensemble using the previously defined parameters for pressure and temperature for 50 and 1000 ns for ubiquitin and the α-synuclein fragment respectively. The GROMACS 5.1.2 [7] package was used to perform the MD simulations, whereas VMD 1.9.1 and Tachyon [8, 9] and UCSF Chimera packages were used to visually analyze the trajectories and render images respectively. Chimera is developed by the Resource for Biocomputing, Visualization, and Informatics at the University of California, San Francisco (supported by NIGMS P41-GM103311).

We received a trajectory of dense solution of 8 villin headpiece chains from Michael Feig et al. [10] for analysis with CONAN. It was obtained with CHARMM [11] through OpenMM. The solution has an effective density of 32 mM, the villins were neutralized with Cl^- ions. The CHARMM 36 force field was used for it with TIP3P water and an increased water-protein interaction by a factor of 1.09 [12]. The simulation was performed in the NVT ensemble with a cubic box with a side of about 7.4 nm. The trajectory has a length of 2μs using a 2 fs time step and a Langevin thermostat with a friction coefficient of 0.01 ps^{-1}. The particle-mesh Ewald method was used for Coulomb interactions.

1.2 Force-probe molecular dynamics simulations of ubiquitin

The molecule of ubiquitin in the conformation extracted at the end of the equilibrium simulation, was placed in a rectangular box of dimensions 28×7×7 nm for force-probe MD simulations, where the centers of mass of the N- and C-terminal residues were aligned with the longest semi-axis. The protein was placed at the center of the box and after the NVT and NpT equilibration steps mentioned above, it was pulled at its N- and C-termini with the first and the last residues being considered as pulling groups. Center of mass pulling was performed on these groups by applying a harmonic potential to achieve constant velocity pulling at the speed of 0.1 ms^{-1} and a force

constant of 500 kJ $mol^{-1}nm^{-2}$. The pulling was directionally applied along the longest semi-axis of the box and in opposite directions for the two pulling groups. The simulations were stopped after 235.6 ns, when secondary structure elements of ubiquitin were fully lost.

1.3 CONAN output and specific methods

1.3.1 Basic output

The output of CONAN is a set of plain-text files and images. The advantage of plain-text over more compact forms is the ease of a quick comparison and filtering right in the terminal (through tools like awk, sort, diff, grep).

1.3.2 Further classes of output

A number of advanced features are available in CONAN: principal component analysis [13], hierarchical clustering of contact maps (classifying frames from a trajectory), hierarchical clustering of residues (classifying residues and detecting domains), and blocking analysis [14] (detecting the correlation time of contact maps). The same classes of analysis are also available for the analysis of asymmetric data, i.e., contacts formed between chains or domains. In Section 6, we show an analysis of protein assembly as an example.

1.3.3 Correlation contact maps

CONAN can compute four kinds of correlations to extract dynamical information (PCC(A(t), B(t)) is the Pearson correlation coefficient between vectors A and B, commonly known as the "r-value"):

PCC($r_{ij}(t)$, t) : time-correlation of inter-residue distance, identifying drifts.

PCC($r_{ij}(t)$, A(t)) : correlation of inter-residue distance and observable A, identifying sequence determinants.

PCC($r_{ij}(t)$, $r_{ab}(t)$) : correlation of inter-residue distance and a specific distance between a pre-defined pair of residues a and b. This is in the "zoom" module. Any number of such pairs ab can be specified.

PCC($N_i(t)$, $N_j(t)$) : inter-residue cross-correlation of the weighted number of contacts $N(t)$.

The "weighted number of contacts" is defined as:

$$N_i(t) = \sum_j (1 - \frac{r_{ij}(t)}{r_{\text{cut}}}).$$

This cross-correlation is a fitting-free alternative to the commonly used "dynamic cross correlation" of deviations from average positions. Note that all of the vectors have the same length, which is the number of frames in the simulation: "missing values", i.e., the distance between two residues that move outside the main cutoff radius, are replaced by the main cutoff radius.

1.4 Hierarchical clustering

CONAN uses Ward's agglomerative hierarcical clustering algorithm [15] to divide residues of a protein or frames of a trajectory into clusters, each with a representative data point (residue or frame) called medoid. Users can opt to interactively choose the desired number(s) of clusters, or to pre-define this number. The dendrogram, for ease of visibility, only displays the top 100 nodes if there are more than 100 data points. The medoid is defined after the clustering is complete by finding the data point with the lowest average distance to data points in its own cluster.

1.4.1 Frames

For the clustering of frames (conformations) composing a trajectory, we use the inter-frame RMSD as the distance metric. Between a conformation at t_1 and t_2, the distance will be given by:

$$\text{RMSD}(t_1, t_2) = \frac{1}{N_{\text{res}}} \sum_{i,j} \Delta r_{ij}(t_1, t_2)^2.$$

1.4.2 Residues

For the clustering of residues, we use the following three metrics between two residues i and j:

1. Average distance in real space:

$$d_{ij} = \bar{r}_{ij}$$

This is a straightforward criterion that is however still a better choice than the distance in the average structure since it does not involve a fitting procedure.

2. Total interaction time:

$$d_{ij} = 1 - \frac{t_{ij}^{\text{inter}}}{t^{\text{total}}}$$

where we use the interaction time between i and j and subtract this from 1 to have high numbers corresponding to low distances. This can be similar to the quantity calculated in Eq. 1, but it can be more representative of actual physical interactions.

3. Inter-residue cross-correlation:

$$d_{ij} = 1 - \text{PCC}(N_i(t), N_j(t))^2,$$

where $\text{PCC}(N_i(t), N_j(t))$ has been described before (Section 1.3.3). This correlation is squared to ameliorate the effect of spurious correlations.

1.5 Principal component analysis

CONAN can be used to do principal component analysis (PCA) on inter-residue distances. Therefore, it uses internal rather than Cartesian coordinates to perform a principal component analysis. Interestingly, the use of internal versus Cartesian coordinates has been recently investigated showing that even though Cartesian coordinates may capture overall motions of a protein, internal coordinates more accurately catch the underlying free-energy landscape of protein dynamics [13].

PCA is implemented across all the residue pairs that ever come closer than the main cutoff. Supposing that there are M such residue pairs, where $M \leq (N_{\text{res}}(N_{\text{res}} - 1)/2)$ if there are N_{res} residue pairs, CONAN computes and diagonalizes the $M \times M$ covariance matrix and prints out a user-defined number of eigenvectors and projections in order of eigenvalues. The time evolution of a residue pair distance will then be given by:

$$r_{ij} = \bar{r}_{ij} + \sum_{u}^{M} c_u^{ij} x_u(t) \approx \bar{r}_{ij} + \sum_{u}^{M_{\text{cut}}} c_u^{ij} x_u(t),$$

where the last approximation shows the often-used truncation of only the first few (in this example, M_{cut}) projections, c_u^{ij} denote the coefficients of principal component u on residue pair ij, and $x_u(t)$ is the time evolution of the projection onto principal component u.

1.6 Asymmetric contact maps

CONAN can do the above analyses on asymmetric cases, i.e., monitoring contacts between one range of residues and another one. This can be a basis for analyzing an interaction between domains or between protein-protein chains.

We will analyze villin-villin interchain docking in Section 6. In the special case of dimeric docking, such as this one, CONAN also takes into account the fact that the order of chains is arbitrary. It sorts dimers by preferring poses in which the N-terminus of the first chain is closer to the C-terminus of the second chain. It does this by comparing the "weighted number of contacts" in both the upper and lower triangles of the inter-chain contact map, and transposing the contact map if the lower triangle is found to have shorter distances than the upper one.

2 Equilibrium simulations: ubiquitin

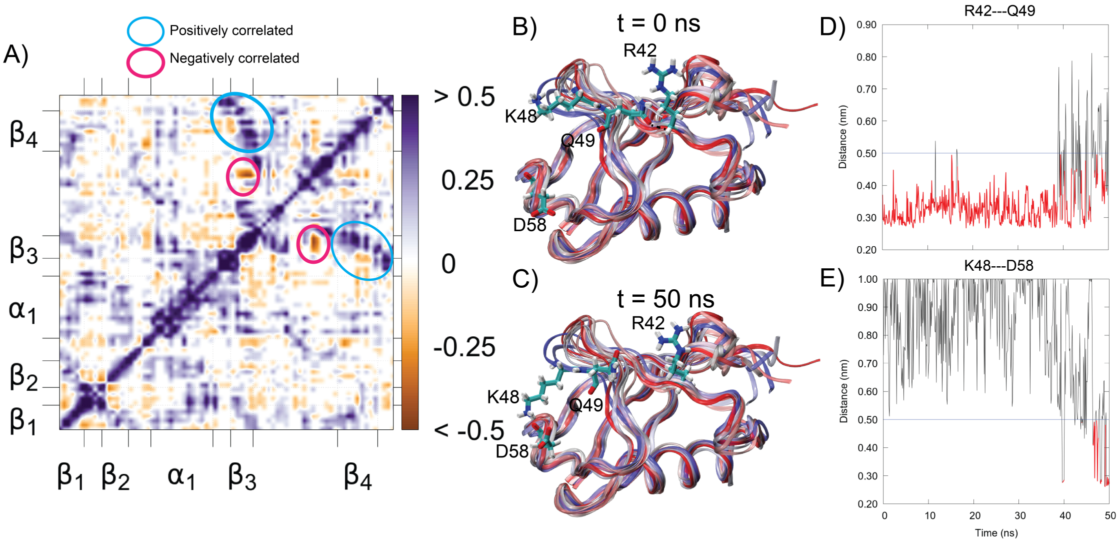

Figure S1: Inter-residue cross-correlation reveals competitive interactions. (A) Inter-residue cross-correlation between the weighted number of contacts of residues in ubiquitin. Particularly low and high values are highlighted in pink (negative cross-correlation, i.e., competitivity) and blue (positive cross-correlation, i.e., cooperativity). The pink circle highlights a competitivity between R42-D58, despite the fact that these residues are well outside each other's radii of interactions. This can be explained by the breaking of a contact between R42-Q49 (shown at t=0 ns in (B) and the time evolution is shown in D) and the forming of a salt bridge between K48-D58 (shown at t=50 ns in C and the time-evolution shown in E).

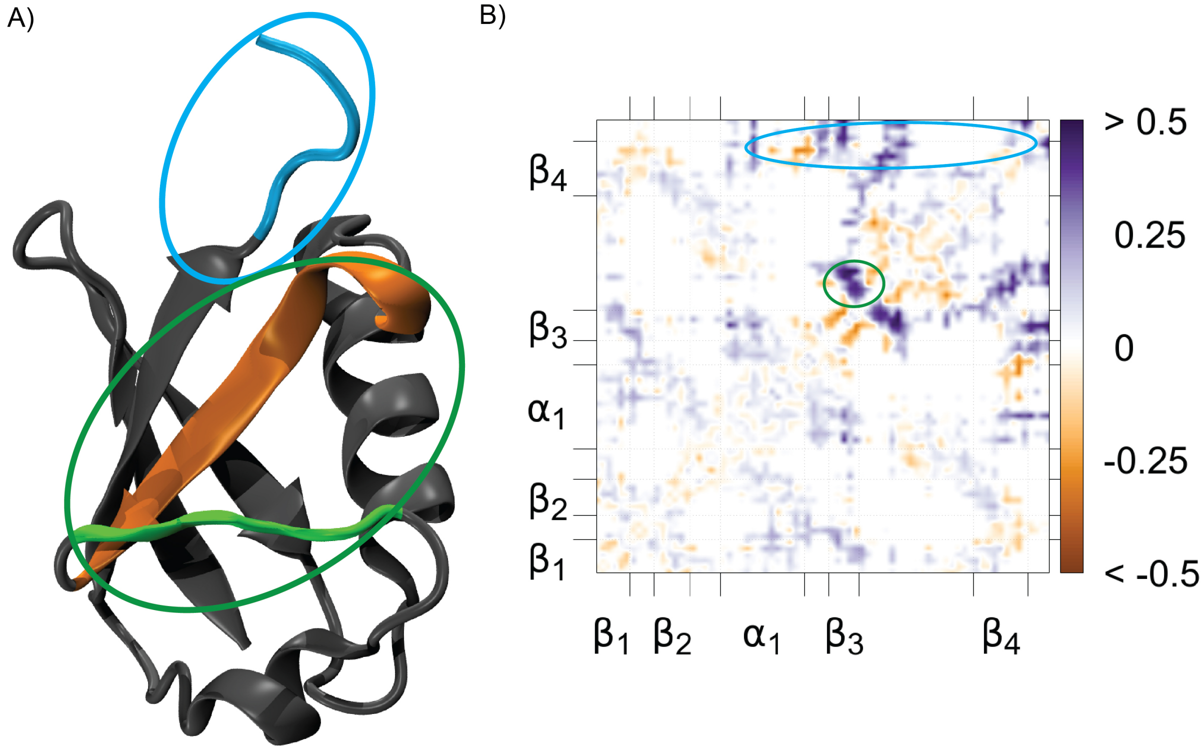

Figure S2: Pearson's correlation of the contact distances with radius of gyration sampled for ubiquitin. (A) Structure of ubiquitin highlighting the regions that show the highest positive correlation between inter-residue distances and the radius of gyration sampled during the simulations. (B) The symmetric correlation map shows positive and negative correlation (Pearson's correlation coefficient) in purple and orange respectively. there are two major regions of contacts that explain increases in the radius of gyration. Firstly, whenever the C-terminal flexible linker loses contacts with the rest of the protein, this increases the radius of gyration, and secondly, the distances between residues in the flexible linker and strand β_3 also positively correlate with R_g. In both cases, the correlation involves the distancing of a flexible linker from the rest of the protein structure, i.e., the average distances from the center of mass of the entire structure will tend to increase, thereby increasing the radius of gyration.

3 Unfolding simulations: ubiquitin

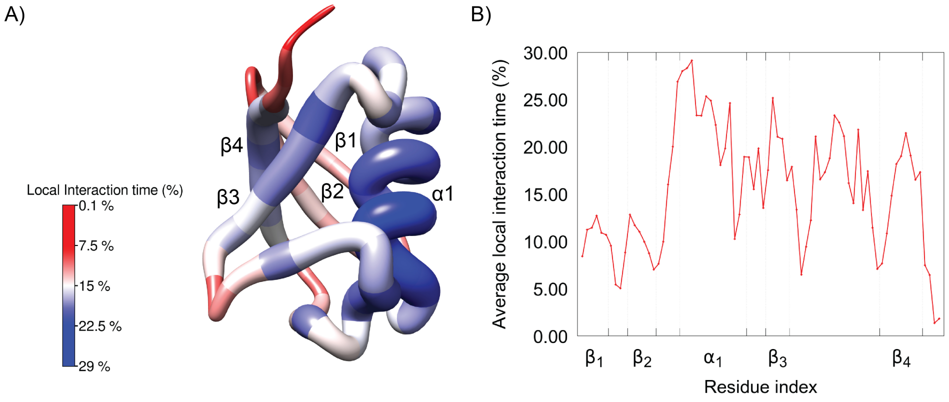

Figure S3: Local interaction time calculated for the unfolding trajectory of ubiquitin. (A) Structure of ubiquitin showing, qualitatively, the local interaction lifetime measured in the unfolding pathway that is quantitatively shown in (B) as a function of the protein's sequence. The structure has been colored according to the "local interaction time". The radius of the ribbons is proportional to this observable, quantitatively reported in the color palette on the left hand side of the figure. Regions in red correspond to parts of the protein that have the longest-lived interactions, i.e., unfold last.

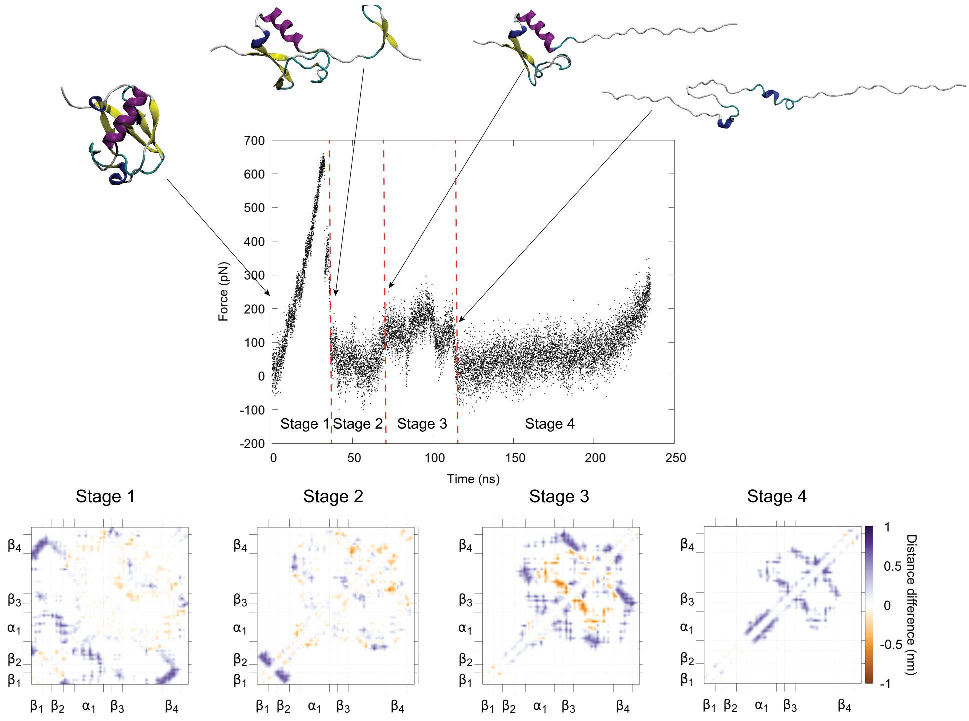

Figure S4: Force-driven unfolding of ubiquitin analyzed through differential contact maps. The force profile as a function of time of ubiquitin is shown in the top panel and divided into four distinct stages (Stages 1 to 4) following clear trends in the evolution of the force withstood by the molecule. For each stage, representative conformations are shown using a cartoon representation and colored by secondary structure. α-helices are colored purple, 3-10 helices blue, β-strands yellow, β-turns cyan, and loops white. In the lower panel, differential contact maps for each stage showing the distance differences for formed (orange) or broken (purple) contacts are shown.

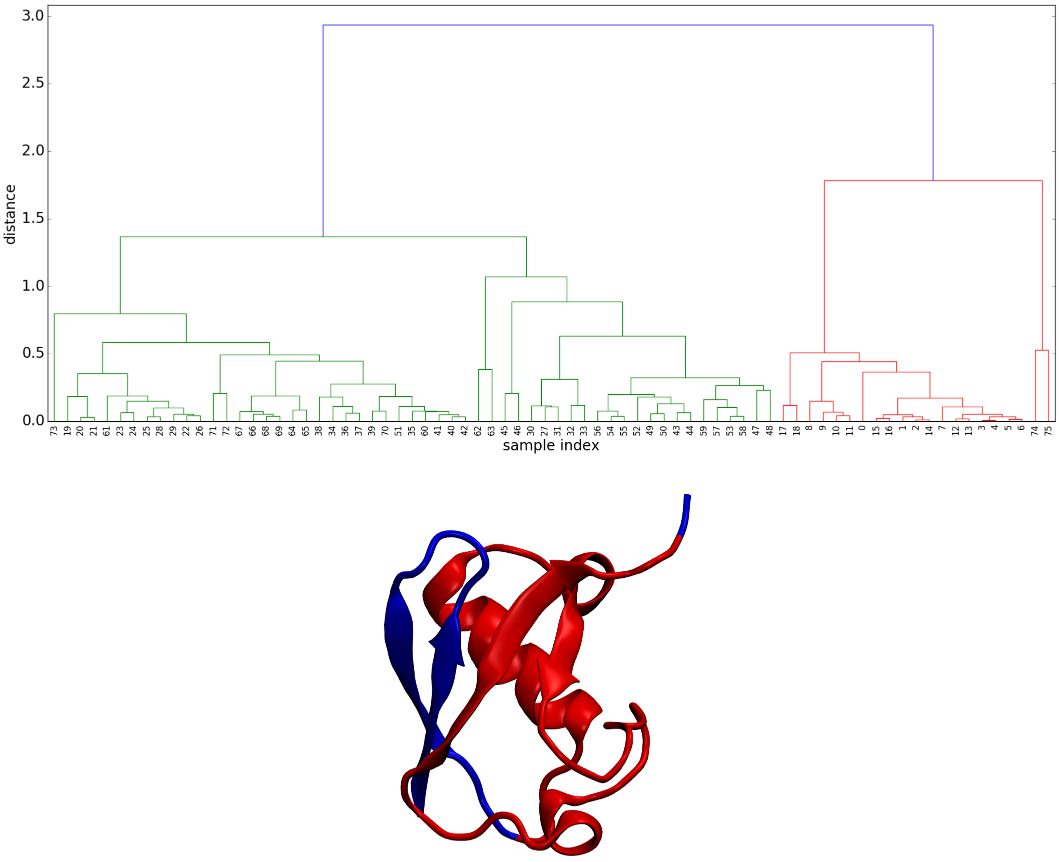

Figure S5: Top: The dendrogram of ubiquitin residues when clustering based on inter-residue cross-correlation. Bottom: The structure of ubiquitin colored by a two-cluster division based on the above dendrogram. The first 19 residues, forming the hairpin β_1-β_2, form one cluster along with the last two residues (74-75). This is because all of these residues had correlated contact losses in the first part of the trajectory. The internal 55 residues (19 to 73) unfolded later, therefore they belong to the other cluster.

4 Equilibrium simulation on α-synuclein fragment and E46K mutant

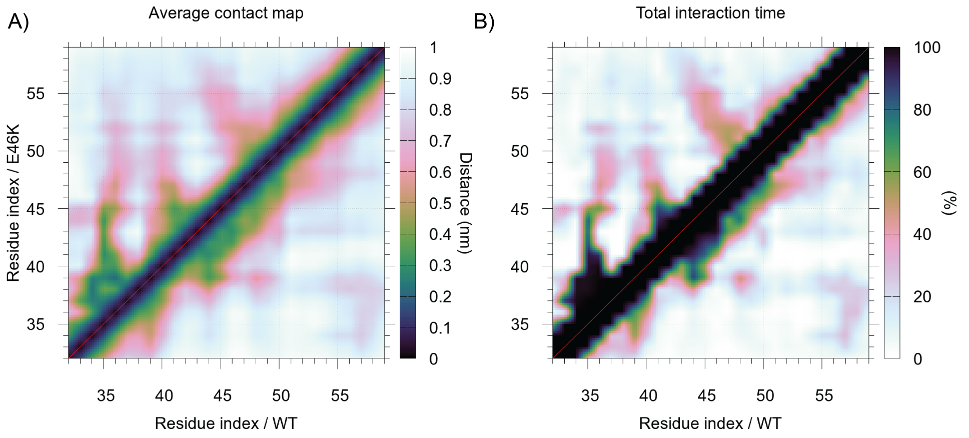

Figure S6: Conformational dynamics of a wild-type and E46K mutant of an α-synuclein fragment investigated through the analysis of contacts. (A) Asymmetric average contact map for the wild-type (lower triangle) and E46K mutant (upper triangle) of an α-synuclein molecule calculated by CONAN. (B) Total interaction time as calculated by CONAN for both the simulated systems (WT and E46K are shown in the lower triangles and upper triangles respectively).

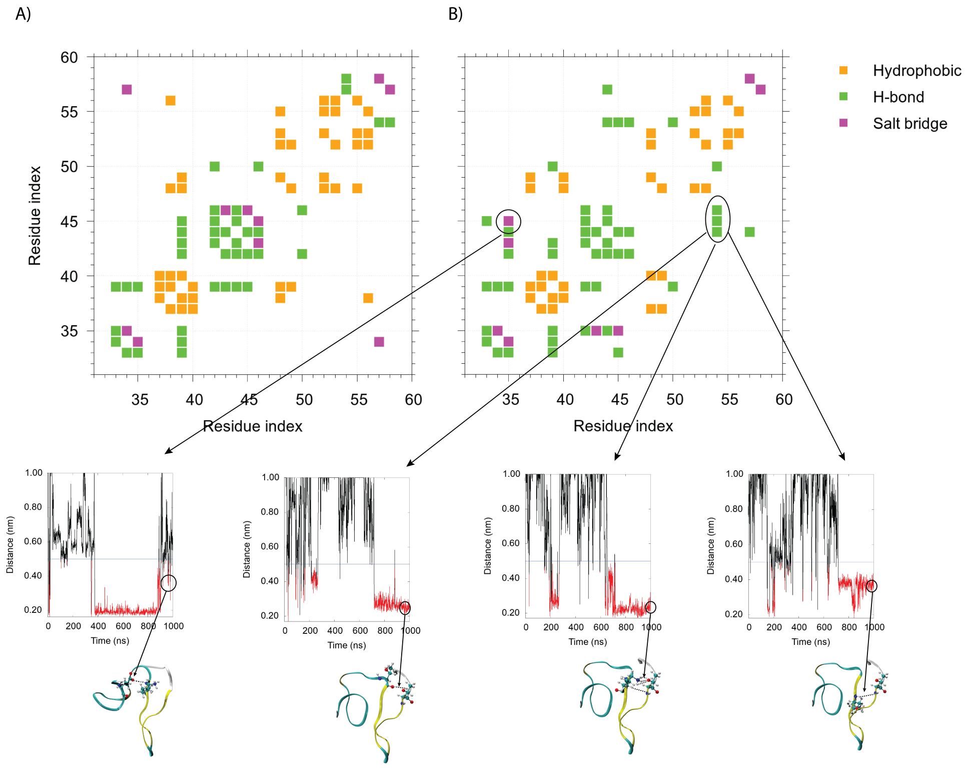

Figure S7: Classification of interaction types performed by CONAN. (A) and (B) show the contact maps colored according to the interaction types occurring within the simulated molecules for the WT (A) and E46K mutant (B) of the simulated α-synuclein molecules. The interaction network mainly responsible for the α to β conformational switch are circled and molecular distances as a function of simulated time are shown in the panel below and drawn in black or red when they are above or below the 0.5 nm cutoff respectively.

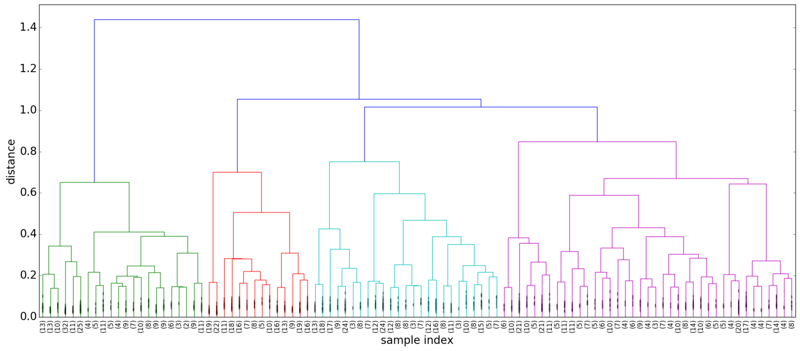

Figure S8: Dendrogram of the α-synuclein fragment (wild type) trajectory.

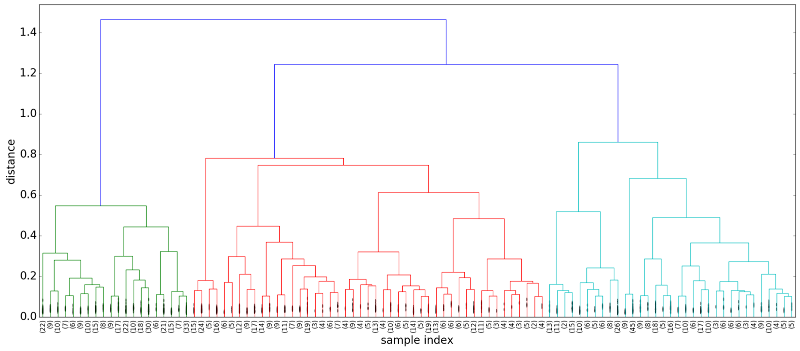

Figure S9: Dendrogram of the α-synuclein fragment (E46K mutant) trajectory.

5 Clustering non-equilibrium pathways: desmoplakin

CONAN can classify *pathways* rather than frames or residues. To this end, we will use the desmoplakin unfolding trajectories from Ref. [16]. This clustering could be done by linking the python executable as a library, but here, we will illustrate this doing the following method:

1. Run a separate CONAN run on each unfolding trajectory $n = 1..N_{\mathrm{traj}}$.
2. Define an inter-residue "distance" based on $d_{ij}(n) = 1 - t^{ij}_{\mathrm{lifetime}}$, where $t^{ij}_{\mathrm{lifetime}}$ is obtained from point 1.
3. Create a fictitious trajectory of N_{traj} frames, where this d_{ij} acts as a distance and n is a time.
4. Cluster this fictitious trajectory. The resulting classification will characterize the unfolding pathways.

Based on the obtained dendrogram (Fig. S10), we could reasonably choose two or three clusters. For simplicity, we choose two of them.

The average "contact maps" of the two clusters of trajectories are shown in Fig. S11. The main difference between the two clusters is the relatively earlier unfolding of helix 5C.

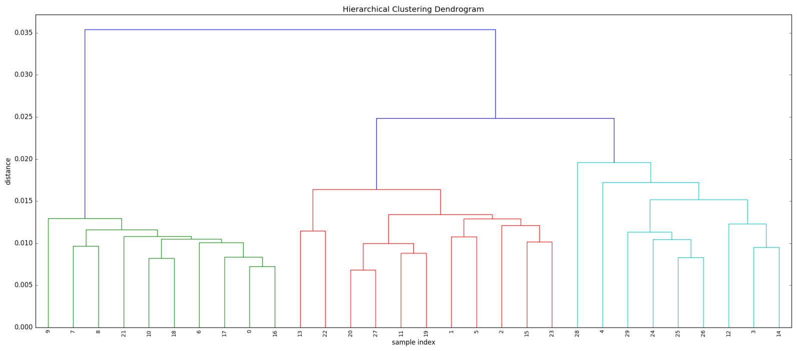

Figure S10: Classification of the desmoplakin unfolding trajectories from Daday et al, Sci Rep 2017. Indices from 0-9 refer to unfoldings at v=1.0 m/s, 10-19 to those at v=1/3 m/s, and 20-29 to those at v=1/10 m/s.

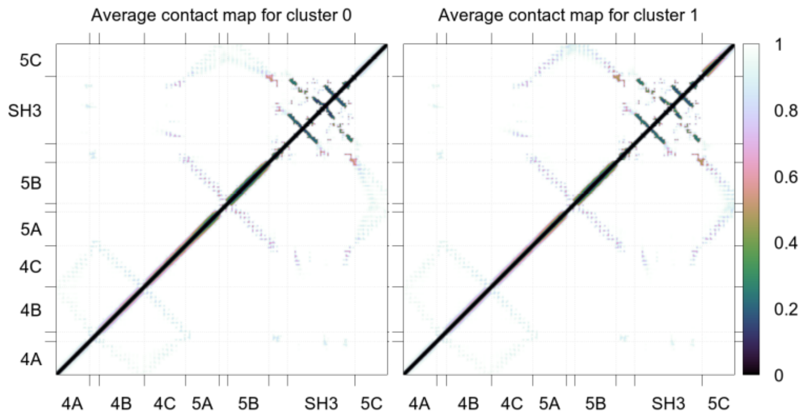

Figure S11: Characteristics of the two clusters of unfolding trajectories for desmoplakin. The shown quantity is 1-(interaction lifetime) computed on the unfolding trajectories.

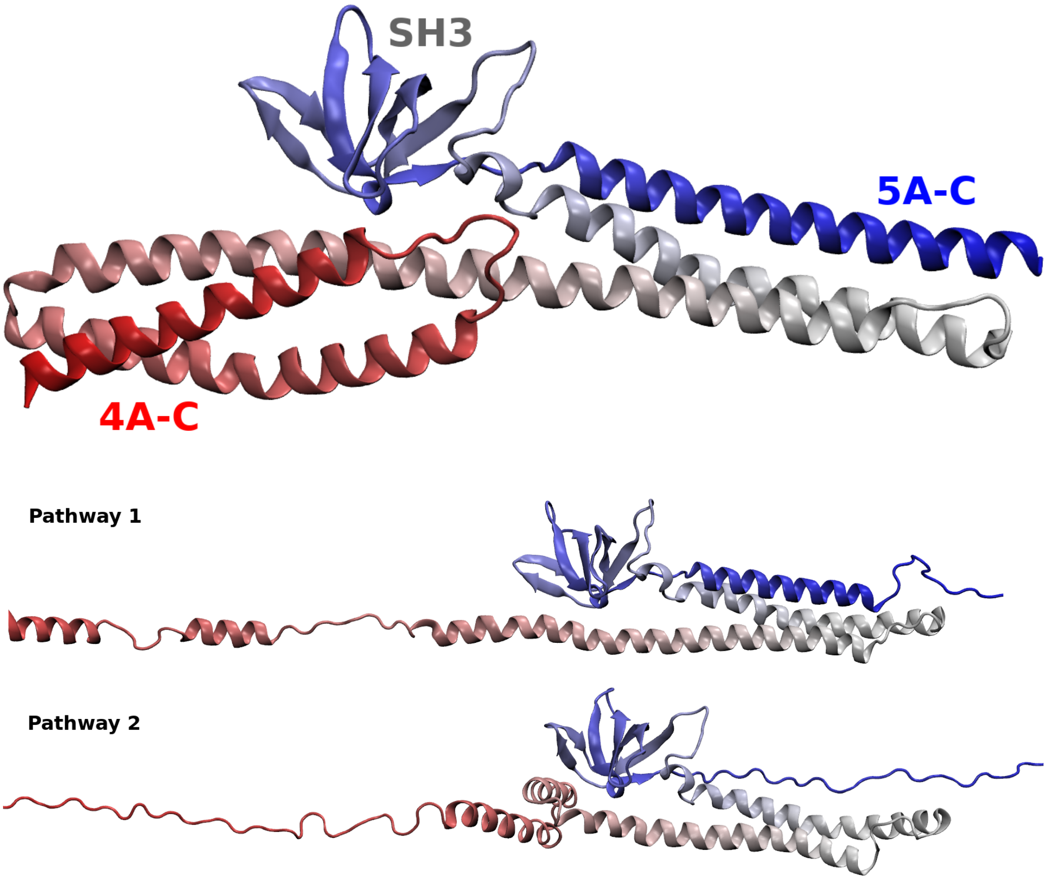

Figure S12: Top: Initial structure of spectrin repeats 4-5 of desmoplakin, consisting of helices 4A-4C, (red to gray) 5A-5C (gray to blue) and an SH3 domain insertion. Bottom: Partially unfolded states in the central two unfolding simulations of desmoplakin. Both correspond to a relative extension of about 20 nm. Helix 5C is fully unfolded in pathway 1 and almost fully intact in pathway 2.

6 Asymmetric contact maps: villin headpiece docking

We analyzed a sample trajectory containing 8 villin headpiece domains in a cubic box. This trajectory was kindly provided by Michael Feig's group, related to Ref. [10]. The trajectory is 2μs long, sampled every 100p̃s (20000 frames).

We show here CONAN's ability to analyze inter-chain interactions. There are 8 chains, so there are a total of 28 possible pairs of chains. We proceeded as follows:

1. We analyzed the trajectory using the 8 chains as "residues" and considering only heavy atom interactions. The 28 possible pairs are considered to interact with cutoffs of $r_{\text{inter}} = 3$ Å to form and $r_{\text{inter}}^{\text{high}} = 5$ Åto break. An example time-evolution plot is in Figure S13.

2. Out of each of the 28 pairs, we extract interactions which last at least 10 ns. We obtain contact maps from these interactions once every 1 ns. This defined a total of 188 interactions and 8376 "binding poses". Each of the 28 pairs of chains formed at least 2 interactions during the simulations.

3. We perform a hierarchical clustering of these 8376 binding poses.

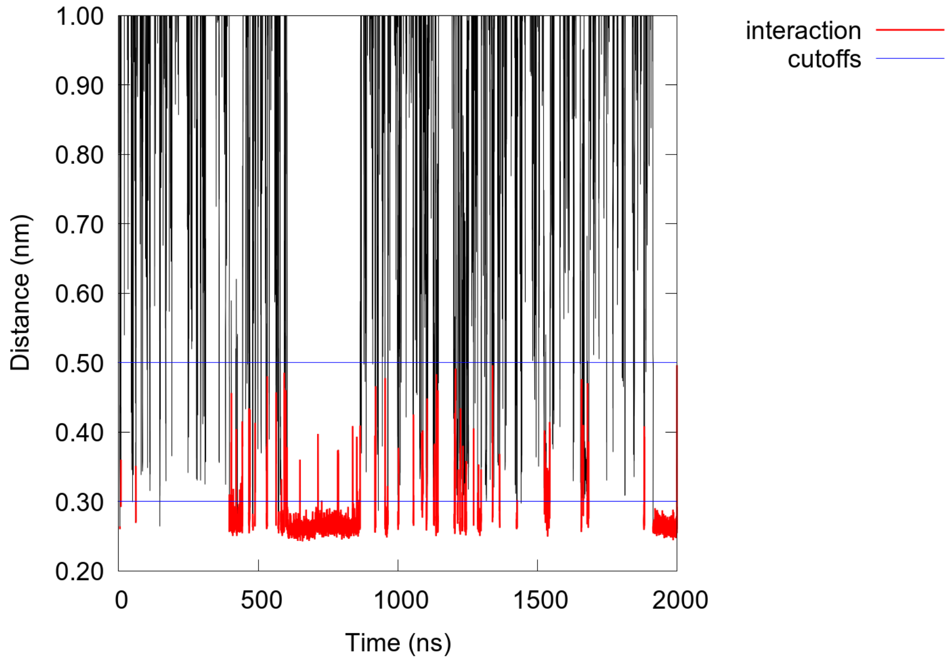

Figure S13: Inter-chain distance between two villin headpiece molecules. Interactions form at any inter-heavy atom distance 3Åand break at 5Å. Only one example plot is shown, the initial screening is based on 28 such inter-chain distance plots.

Based on the dendrogram in Fig. S14, we choose 3 clusters and analyze them further.

Cluster 0 contains 3099 frames, and the most characteristic interactions are salt bridges between R15 and D6 (23% between chains A-B and 15% between chains B-A). We show the overall lifetimes of interactions between the two chains as well as an example 3D snapshot (in which both copies of R15 are involved) in Fig. S15.

Cluster 1 contains 795 frames, and it only occurred once in the samples. Given that it lasted almost 800 ns, it represents an exceptionally strong docking pose. The example snapshot in Fig. S16 shows how one of the villin headpiece chains partially unfolds and the two chains form a strong hydrophobic packing.

Cluster 2 contains 4482 frames, and it is most well characterized by a hydrophobic packing between M1, L2 at the N-terminus of one chain and L35, F36 at the C-terminus (see Fig. S17) of the other. These interactions occurred about 15-20% of the frames in the clusters.

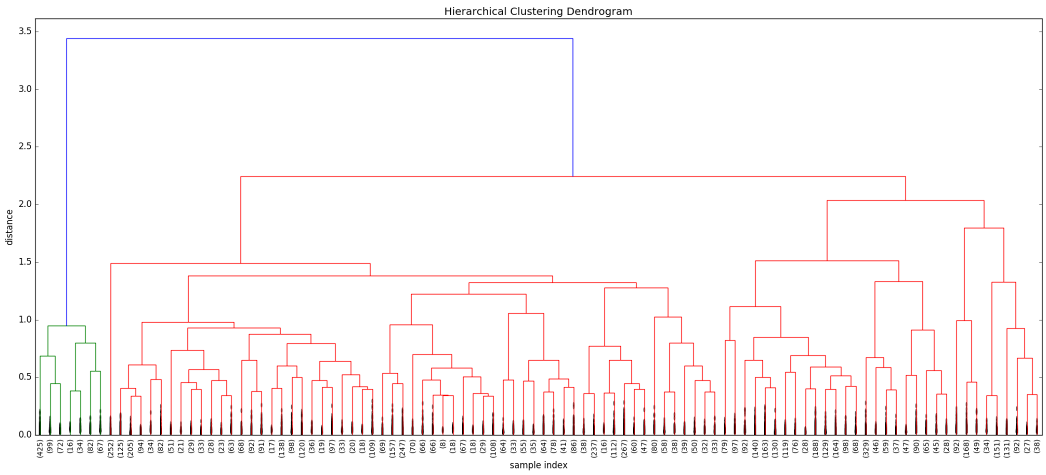

Figure S14: The top 100 nodes of the villin-villin docking dendrogram. Based on this dendrogram, we choose 3 clusters and analyze them in the following.

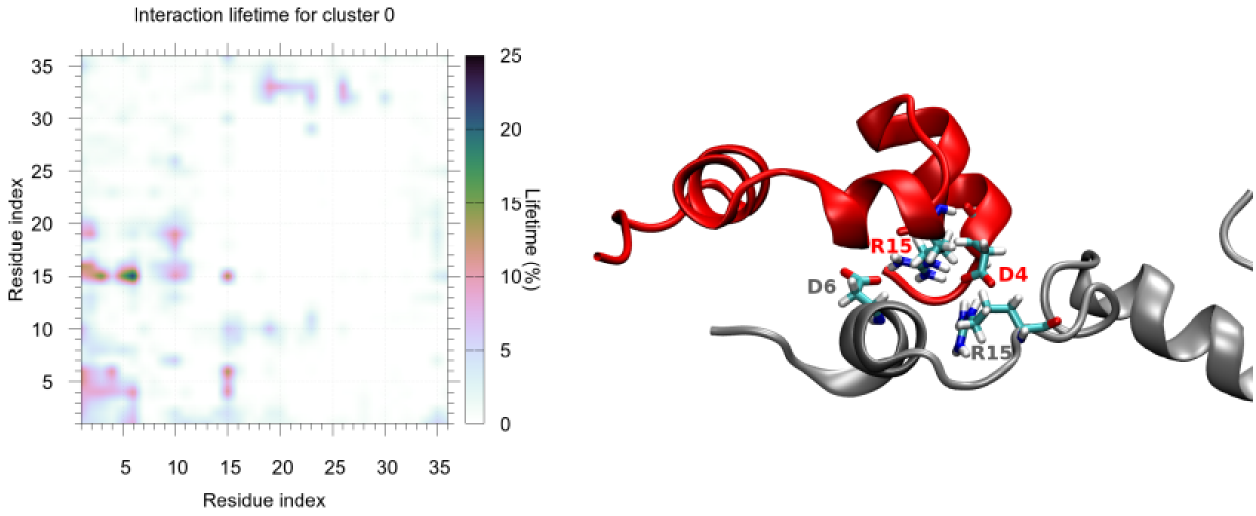

Figure S15: Cluster 0 of villin-villin docking poses. Left: inter-chain interaction lifetime characterizing the interaction. Right: snapshot illustrating the salt bridge R15-D6. In this case, R15 of the other chain also forms a salt bridge, with D4.

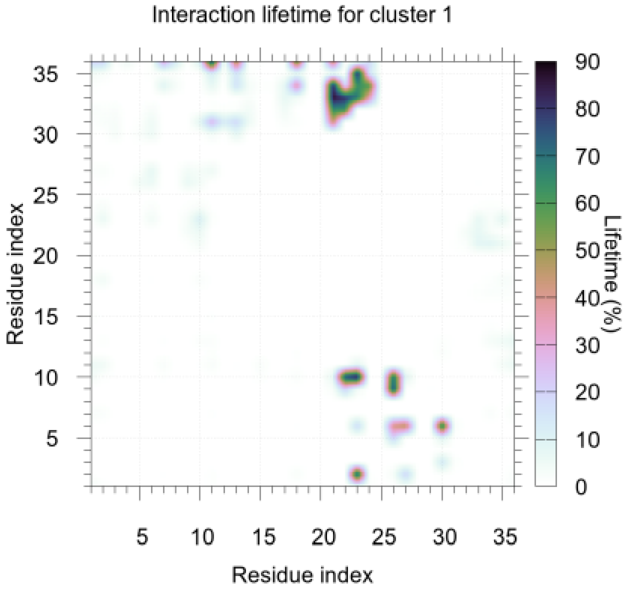

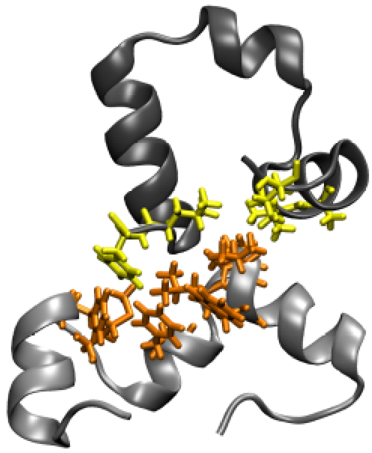

Figure S16: Cluster 1 of villin-villin docking poses. Left: inter-chain interaction lifetime characterizing the interaction. Right: snapshot illustrating the hydrophobic docking between the chains. All hydrophobic residues which are at most 5Å from the other chain are shown.

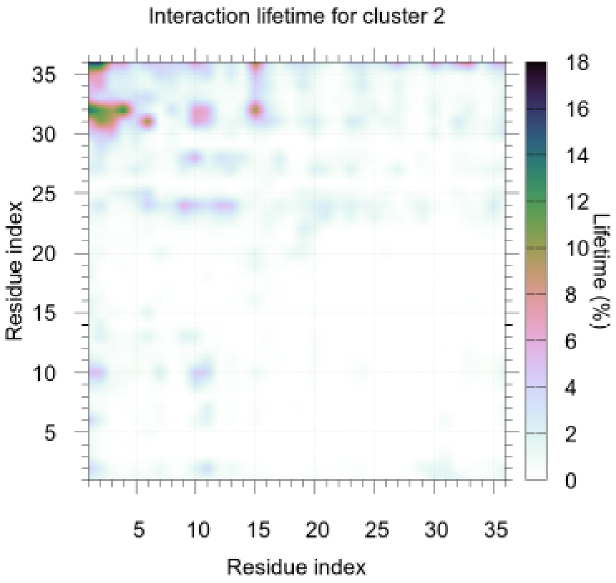

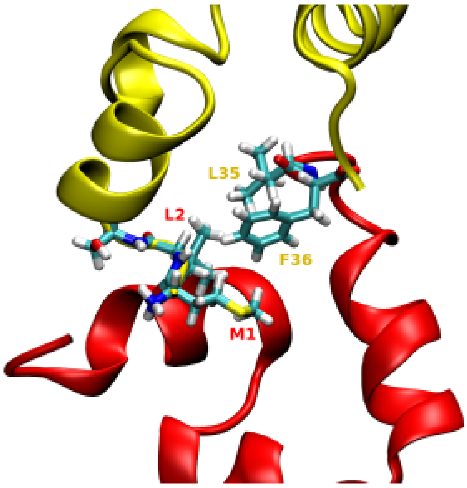

Figure S17: Cluster 2 of villin-villin docking poses. Left: inter-chain interaction lifetime characterizing the interaction. Right: snapshot illustrating the hydrophobic docking between the N-terminus of one chain and the C-terminus of the other.

7 All input files used in the writing of the main article

7.1 Ubiquitin equilibrium simulation

```
TRAJ md.xtc
COORD md.tpr
NLEVEL 1001
TRUNC  1.0
DT 100
TRUNC_INTER 0.5
GNUS_PATH ~/CONAN/input_gnuplotscripts/
DOMAINS domains.txt
PEARSON_TIME yes
PEARSON_OBS area_gyrate_100ps.txt
COORD_PDB ubi.pdb
ZOOM_LIST zoom_list.txt
```

7.2 Ubiquitin unfolding simulation

```
TRAJ pull.xtc
COORD pull.tpr
NLEVEL 1001
TRUNC  1.0
DT 100
TRUNC_INTER 0.5
GNUS_PATH ~/CONAN/input_gnuplotscripts/
K_RES_CLUSTERS 1-5
DOMAINS domains.txt
COORD_PDB ubi.pdb
```

7.3 α-synuclein fragment and E46K equilibrium simulations

```
TRAJ pull.xtc
COORD pull.tpr
NLEVEL 1001
NTERM 32
TRUNC  1.0
DT 100
TRUNC_INTER 0.5
K_TRAJ_CLUSTERS 2-5
GNUS_PATH ~/CONAN/input_gnuplotscripts/
```

7.4 Comparison between α-synuclein fragment wild type and E46K mutant

This is used by the secondary CONAN tool, conan.comp.py.

```
RUN_A ../WT
RUN_B ../E46K
TITLE_A "wildtype"
TITLE_B "E46K"
```

7.5 Desmoplakin clustering of lifetimes

For each of the 30 unfoldings, we used the following CONAN input:

```
TRAJ pull.xtc
COORD pull.tpr
NLEVEL 1001
NTERM 32
TRUNC  1.0
DT <deltat>
TRUNC_INTER 0.6
GNUS_PATH ~/CONAN/input_gnuplotscripts/
```

where deltat=0.25 nm/v (e.g., for $v = 1$ m/s, we take snapshots every 250 ps). This means that every pulling velocity contributes the same number of frames in the full contact analysis. Then, we created a file where we print $d_{ij} = 1 - t_{\mathrm{life}}$ (t_{life} is contained in the file lifetime.dat).

Then, using CONAN's REREAD feature, we read these 30 "frames" and cluster them:

```
REREAD .
TRUNC  1.0
K_TRAJ_CLUSTERS 2-3
GNUS_PATH ~/CONAN/input_gnuplotscripts/
```

7.6 Villin headpiece docking

We began by running CONAN on the entire trajectory, uniting all atoms from each protein as to have the same residue ID. We used this file (sys_chains.gro) as a structure file for the contact analysis. all.txt contains all 28 possible chain pairs.

```
TRAJ traj.gro
COORD sys_chains.gro
NLEVEL 1000
TRUNC 1.00
TRUNC_INTER 0.3
TRUNC_INTER_HIGH 0.5
GNUS_PATH ~/CONAN/input_gnuplotscripts/
RUN_MDMAT yes
ZOOM_LIST all.txt
```

The output of CONAN in the "zoomed in" version was next used to generate interesting fragments of the simulation time (any time at least 10 ns long where the interaction between the chains is uninterrupted), using simple awk commands. For each of these interesting time fragments, CONAN was used on the particular 72-residue fragment.

```
TRAJ traj.gro
COORD sys_chains.gro
NLEVEL 1000
TRUNC 1.00
INDEX index.ndx
TRUNC_INTER 0.5
TRUNC_INTER_HIGH 0.5
BEGIN <begin>
END <end>
GNUS_PATH ~/CONAN/input_gnuplotscripts/
RUN_MDMAT yes
ZOOM_LIST all.txt
```

7.7 Supplementary videos

- Supplementary Video V1: ubiquitin unfolding (framewise contact maps).
- Supplementary Video V2: ubiquitin unfolding (framewise differential contact maps w.r.t. initial frame).
- Supplementary Video V3: ubiquitin unfolding (framewise differential contact maps w.r.t. previous frame).
- Supplementary Video V4: α-synuclein fragment (wild type).
- Supplementary Video V5: α-synuclein fragment (E46K mutant).

References

[1] R. Ramage, J. Green, T. W. Muir, O. M. Ogunjobi, S. Love, and K. Shaw, "Synthetic, structural and biological studies of the ubiquitin system: the total chemical synthesis of ubiquitin.," *Biochem. J.*, vol. 299, pp. 151–158, 1994.

[2] E. A. Mirecka, H. Shaykhalishahi, A. Gauhar, Ş. Akgül, J. Lecher, D. Willbold, and M. Stoldt, "Sequestration of a β-hairpin for control of α-synuclein aggregation.," *Angewandte Chemie International Edition*, vol. 53, pp. 4227–4230, 2014.

[3] Y. Duan, C. Wu, S. Chowdhury, M. C. Lee, G. Xiong, W. Zhang, R. Yang, P. Cieplak, R. Luo, T. Lee, J. Caldwell, J. Wang, and P. Kollman, "A point-charge force field for molecular mechanics simulations of proteins based on condensed-phase quantum mechanical calculations.," *J. Comp. Chem.*, vol. 24, pp. 1999–2012, 2003.

[4] W. L. Jorgensen, J. Chandrasekhar, and J. D. Madura, "Comparison of simple potential functions for simulating liquid water.," *J. Chem. Phys.*, vol. 79, pp. 926–935, 1983.

[5] G. Bussi, D. Donadio, and M. Parrinello, "Canonical sampling through velocity rescaling," *J. Chem. Phys.*, vol. 126, p. 014101, 2007.

[6] M. Parrinello and A. Rahman, "Polymorphic transitions in single crystals: A new molecular dynamics method.," *J. Appl. Phys.*, vol. 52, pp. 7182–7190, 1981.

[7] D. Van Der Spoel, E. Lindahl, B. Hess, G. Groenhof, A. E. Mark, and H. J. C. Berendsen, "GROMACS: Fast, flexible, and free.," *J. Comp. Chem.*, vol. 26, pp. 1701–1718, 2005.

[8] W. Humphrey, A. Dalke, and K. Schulten, "VMD: Visual molecular dynamics," *J. Mol. Graph.*, vol. 14, pp. 33–38, 1996.

[9] J. E. Stone, "An efficient library for parallel ray tracing and animation," Master's thesis, 1998.

[10] G. Nawrocki, P. Wang, I. Yu, Y. Sugita, M. Feig, *et al.*, "Slow-down in diffusion in crowded protein solutions correlates with transient cluster formation," *J. Phys. Chem. B*, vol. 121, pp. 11072–11084, 2017.

[11] B. Brooks, C. Brooks, III, A. MacKerell, Jr, L. Nilsson, R. Petrella, B. Roux, Y. Won, G. Archontis, C. Bartels, S. Boresch, A. Caflisch, L. Caves, Q. Cui, A. Dinner, M. Feig, S. Fischer, J. Gao, M. Hodoscek, W. Im, K. Kuczera, T. Lazaridis, J. Ma, V. Ovchinnikov, E. Paci, R. W. Pastor, C. B. Post, J. Z. Pu, M. Schaefer, B. Tidor, R. M. Venable, H. L. Woodcock, X. Wu, W. Yang, D. York, and M. Karplus, "CHARMM: The biomolecular simulation program," *J. Comp. Chem.*, vol. 30, pp. 1545–1614, 2009.

[12] R. Best, W. Zheng, and J. Mittal, "Balanced protein-water interactions improve properties of disordered proteins and non-specific protein association," *J. Chem. Theory Comput.*, vol. 10, pp. 5113–5124, 2014.

[13] F. Sittel, J. Abhinav, and G. Stock, "Principal component analysis of molecular dynamics: On the use of cartesian vs. internal coordinates," *J. Chem. Phys*, vol. 141, p. 014111, 2014.

[14] H. Flyvbjerg and H. G. Petersen, "Error estimates on averages of correlated data," *J. Chem. Phys.*, vol. 91, p. 461, 1989.

[15] J. H. Ward, Jr., "Hierarchical grouping to optimize an objective function," *J. Am. Stat. Assoc.*, vol. 58, pp. 236–244, 1963.

[16] C. Daday, K. Kolšek, and F. Gräter, "The mechano-sensing role of the unique SH3 insertion in plakin domains revealed by molecular dynamics simulations," *Sci. Rep.*, vol. 7, p. 11669, 2017.
